# Isolation and global occurrence of nitrogen-fixing *Acidobacteriota* in soil environments

**DOI:** 10.1093/ismejo/wrag157

**Published:** 2026-06-18

**Authors:** Hideomi Itoh, Kazumori Mise, Miyu Kuniyasu, Sawa Wasai-Hara, Natsumi Ushijima

**Affiliations:** Biomanufacturing Process Research Center, National Institute of Advanced Industrial Science and Technology Hokkaido, 2-17-2-1 Tsukisamu-higashi, Toyohira-ku, Sapporo, Hokkaido 062-8517, Japan; Biomanufacturing Process Research Center, National Institute of Advanced Industrial Science and Technology Hokkaido, 2-17-2-1 Tsukisamu-higashi, Toyohira-ku, Sapporo, Hokkaido 062-8517, Japan; Institute of Low Temperature Science, Hokkaido University, Kita 19, Nishi 8, Kita-ku, Sapporo, Hokkaido 060-0819, Japan; Department of Biotechnology, Hokkaido High-Technology College, 2-12-1, Megumino-kita, Eniwa, Hokkaido 061-1396, Japan; Biomanufacturing Process Research Center, National Institute of Advanced Industrial Science and Technology Hokkaido, 2-17-2-1 Tsukisamu-higashi, Toyohira-ku, Sapporo, Hokkaido 062-8517, Japan; Graduate School of Dental Medicine, Hokkaido University, Kita 13, Nishi 7, Kita-ku, Sapporo, Hokkaido 060-8586, Japan

**Keywords:** *Acidobacteriota*, nitrogen fixation, soil, isolation, taxonomy, metagenomics

## Abstract

*Acidobacteriota*, one of the most abundant and ubiquitous bacterial phyla in soils, are well recognized for their role in carbon cycling. In contrast, their roles in soil nitrogen cycling remain largely unexplored, although recent metagenome-assembled genome (MAG) analyses suggest that *Acidobacteriota* may harbor genes involved in nitrogen cycling. Here, we provide culture-based evidence of diazotrophy within this phylum and demonstrate the widespread occurrence of nitrogen-fixing *Acidobacteriota* across diverse soil types. From grassland and agricultural soils, we isolated five *Acidobacteriota* strains representing novel taxonomic lineages, four of which harbor functional nitrogenase (*nif*) gene clusters. These strains were capable of fixing atmospheric nitrogen *in vitro* and/or in soil microcosms, as evidenced by acetylene reduction, N_2_-dependent growth, transcription of *nif* genes, incorporation of ^15^N into biomass and soil, and inhibition of nitrogenase activity by ammonium. Furthermore, global-scale meta-analysis of soil metagenomes revealed that *nif*-harboring *Acidobacteriota* are widely distributed and locally dominant across soil types. These results demonstrate the nitrogen-fixing capability of *Acidobacteriota* at the organismal level, complementing MAG-based inferences, and underscore their adaptive capacity in nitrogen-limited environments and their potential contribution to terrestrial nitrogen fixation. We also propose novel taxa within the class *Terriglobia* of the phylum *Acidobacteriota*, including diazotrophic strains, comprising one novel family, three novel genera, and four novel species: *Koromonadaceae* fam. nov., *Koromonas soli* gen. nov., sp. nov., *Koromonas humicola* sp. nov., *Oryzophilus luti* gen. nov., sp. nov., and *Humiphilus diazotrophicus* gen. nov., sp. nov.

## Introduction

The phylum *Acidobacteriota* (formerly *Acidobacteria*) is one of the most ubiquitous bacterial groups in soil microbiomes, with members widely distributed across diverse terrestrial environments—including forest soils, agricultural fields, tundra, and peatlands [1]. In contrast, they are rarely detected in marine environments [2]; this striking difference underscores their identity as a soil-specific bacterial group. Not only are they consistently present across soil environments, but they also often dominate the microbial community; in some soils, *Acidobacteriota*-affiliated 16S ribosomal ribonucleic acid (rRNA) gene reads account for 20%–30% of the total bacterial community [3], and in acidic soils, such as tundra and peatlands, their relative abundance can exceed 60% [4]. Despite their prevalence and dominance, cultured *Acidobacteriota* strains remain far fewer than those of other major soil phyla, such as *Pseudomonadota*, *Bacillota*, and *Actinomycetota* [5]. This cultivation bottleneck continues to limit the experimental characterization of *Acidobacteriota*, constraining a deeper understanding of their physiology and ecological functions.

Members of the phylum *Acidobacteriota* are particularly known for their important role in soil carbon cycling. Physiological and genomic analyses have suggested that many lineages encode diverse carbohydrate-active enzymes, enabling them to degrade plant-derived polysaccharides such as cellulose, hemicellulose, and xylan [6–9]. *Acidobacteriota* have been implicated as key drivers of carbon cycling in highly acidic polar soils and suggested to act as primary degraders of plant litter released by permafrost thaw under global warming scenarios [10]. In addition to carbon cycling, members of this phylum have been reported to participate in iron and sulfur cycling [11–13], utilize trace atmospheric hydrogen [14], perform phototrophy [15], and promote plant growth [16, 17]. Furthermore, several studies have reported the reconstruction and analysis of soil-derived metagenome-assembled genomes (MAGs) of *Acidobacteriota*, suggesting that they may possess additional ecological traits and functions beyond those currently known [18–21]. These limited but compelling findings strongly indicate the ecological importance of *Acidobacteriota* and underscore the urgent need to overcome current limitations in soil microbiology that hinder comprehensive consideration of this ubiquitous and dominant bacterial phylum.

Whereas the involvement of *Acidobacteriota* in carbon and other biogeochemical cycles is increasingly recognized, their role in the nitrogen cycle remains poorly understood. Genomic analyses of cultured *Acidobacteriota* strains have indicated that genes involved in major nitrogen transformation pathways, including nitrification, denitrification, and nitrogen fixation, are largely absent in this phylum [22, 23]. A recent study has provided experimental evidence for N_2_O reduction in *Acidobacteriota*, suggesting their previously unrecognized role in denitrification-related processes [24]. However, with regard to biological nitrogen fixation, the only cultured *Acidobacteriota* strain known to harbor nitrogenase genes (*nif*) required for biological nitrogen fixation is *Holophaga foetida* TMBS4^T^ [22]. Although recent environmental metagenomic studies have detected *nif* genes in MAGs from uncultured *Acidobacteriota* [19–21, 25], and stable isotope analysis based on ^15^N_2_ has also indicated a potential contribution of *Acidobacteriota* to nitrogen fixation in paddy soils [26], no direct experimental evidence from cultured isolates has yet demonstrated functional nitrogen-fixing capacity in this phylum.

In a previous study, we reported the successful isolation of numerous anaerobic *Acidobacteriota* strains from diverse soil and sediment environments, including paddy fields, upland farms, and forest ecosystems [27]. In this study, we extended our isolation efforts and identified several *Acidobacteriota* strains harboring functional *nif* genes in their genomes. We demonstrated nitrogen fixation activity by these strains *in vitro* and under soil conditions and elucidated the global terrestrial distribution of *nif*-harboring *Acidobacteriota* using soil metagenomic datasets. Collectively, these results provide direct experimental evidence from cultured isolates, beyond MAGs-based inference, for biological nitrogen fixation by *Acidobacteriota* and shed light on the underinvestigated role of this soil-dominant phylum in terrestrial nitrogen cycling.

## Material and methods

### Soil sample collection

The soil samples used for isolation were collected (0–3 cm depth) with a sterile spatula from grassland and agricultural fields in Japan (Table S1). After removing plant material and excess surface water, the samples were transported to the laboratory and stored at 4°C until required for use within 3 months. Soil pH (H_2_O) of the isolation source was measured in a soil–water suspension (1:5, w/w) using a pH meter (LAQUAtwin pH-22B, Horiba, Kyoto, Japan). The pH of the soil slurry used for cultivation was measured directly using the same pH meter.

### Isolation of *Acidobacteriota* strains

Screening for nitrogen-fixing *Acidobacteriota* was conducted based on a soil slurry incubation method that has previously enabled the isolation of phylogenetically novel anaerobic diazotrophic lineages within the phyla *Myxococcota* and *Desulfobacterota* [28–35], as well as anaerobic *Acidobacteriota* belonging to the family *Holophagaceae* [27]. Soil samples used as the “medium” were air-dried at 25°C, placed into 5 ml serum bottles, and suspended in distilled water at a soil-to-water ratio of 1:1.5 (w/v). The suspension was pre-incubated at 30°C without shaking for one week to deplete readily available nitrogen sources, such as ammonium, and was then autoclaved at 120°C for 15 min to serve as a sterile soil-based medium (hereafter referred to as soil bottles). Subsequently, 0.05 g of undried soil was added to each soil bottle as the microbial inoculum. The bottles were sealed with butyl rubber stoppers and aluminum caps, and the headspace gas was replaced with N_2_/CO_2_ (80:20, v/v). The bottles were incubated at 30°C without shaking for two weeks. Then, 100 μL of the incubated soil suspension was transferred into soil bottles and incubated for an additional two weeks. This subculturing step was repeated twice in total. The incubated soil slurry was streaked onto 1.5% agar plates of Reasoner’s 2A (R2A) DAIGO (Nihon Pharmaceutical, Tokyo, Japan) supplemented with 5 mM disodium fumarate and 25 μg·ml^−1^ valinomycin to suppress the growth of Gram-positive bacteria. The plates were incubated at 30°C for 7 d under anoxic conditions using the AnaeroPack system (Mitsubishi Gas Chemical, Tokyo, Japan). After isolating single colonies, partial 16S rRNA gene sequences were determined by Sanger sequencing and subjected to similarity searches for preliminary identification following a method described in a previous study [27]. *Acidobacteriota* strains were routinely cultured using R2A agar (BD Difco, Franklin Lakes, NJ, USA) supplemented with 5 mM disodium fumarate (R2Af), as these strains exhibited better growth compared with R2A DAIGO (Nihon Pharmaceutical). Each strain was cultured in R2Af medium and preserved at −80°C with 10% DMSO. The combinations of soil samples used as the slurry medium and microbial inoculum for isolating each strain are summarized in Table S1.

### Genomic analyses of isolated strains

Genomic DNA extraction and sequencing were performed as described in the “Supplementary Information”. The resulting circularized genomes were annotated using the DDBJ Fast Annotation and Submission Tools (DFAST) ver. 1.6.0 [36], which predicted coding sequences (CDSs) with Prodigal, identified tRNA genes using tRNAscan-SE (bacteria), and detected rRNA genes with Barrnap. Functional annotation of the CDSs was performed using DFAST with default parameters. In addition, genes encoding cytochrome c oxidase involved in oxygen respiration and those associated with dissimilatory nitrogen transformation were further verified through the Conserved Domain Database search [37] and KEGG BlastKOALA [38] analyses. Genomic relatedness was evaluated using average nucleotide identity (ANI), digital DNA–DNA hybridization (dDDH), and average amino acid identity (AAI), calculated using JSpeciesWS ver. 4.2.3 [39], DSMZ Genome-to-Genome Distance Calculator (GGDC) ver. 3.0 [40], and FastAAI ver. 0.0.1 [41], respectively. Phylogenomic analysis of the isolates and type strains of the order *Terriglobales* was based on 120 single-copy marker genes. Marker genes were identified using the “classify_wf” command of GTDB-Tk v2.4.1 (default parameters) [42]. The resulting alignment (“gtdbtk.bac120.user_msa.fasta.gz” generated by classify_wf command) was used to construct a maximum-likelihood tree with RAxML-NG v1.2.2 [43] under the WAG model and default settings. Additionally, complete 16S rRNA gene sequences were retrieved from the genomes, and phylogenetic analyses were performed using the maximum-likelihood and neighbor-joining methods, both based on the Tamura-Nei model [44], and node support was evaluated by bootstrap analysis (1,000 replicates), as described previously [45]. The neighbor-joining analysis was conducted to confirm the robustness of the tree topology inferred by the maximum-likelihood method.

### Physiological and chemotaxonomic characterization of isolated *Acidobacteriota* strains

Cell morphology, motility, Gram staining, and spore formation were examined using phase-contrast and transmission electron microscopy following standard staining procedures. Growth characteristics were determined across a range of temperatures, pH values, and NaCl concentrations under anoxic conditions. In addition, oxygen tolerance was assessed under oxic, microoxic, and anoxic conditions. Carbon source utilization, polysaccharide hydrolysis, and enzymatic activities were also assessed. The cellular fatty acids and isoprenoid quinones were analyzed using chromatographic methods. Detailed protocols are provided in “Supplementary Information”.

### Subculture for diazotrophy assessment

To prioritize the assessment of diazotrophic activity, all strains were subcultured in defined minimal media prior to assays. Medium A and Medium B were nitrogen-free minimal media supplemented with 25 mM glucose. Medium A contained (per liter) 0.3 g KH_2_PO_4_, 0.1 g MgCl_2_·6H_2_O, 0.08 g CaCl_2_·2H_2_O, 0.6 g NaCl, 2 g KHCO_3_, 0.02 g MgSO_4_·7H_2_O, 10.7 g 2-(*N*-morpholino)ethanesulfonic acid (MES), 10 ml mineral stock solution [30], and 10 ml vitamin stock solution [30] (pH 5.5). Medium B contained (per liter) 0.25 g KH_2_PO_4_, 0.125 g MgSO_4_·7H_2_O, 0.125 g NaCl, 0.027 g FeCl_3_·6H_2_O, 0.025 g Na_2_MoO_4_·2H_2_O, 0.005 g MnSO_4_·4H_2_O, 0.1 g CaCO_3_, 0.2 mg AlK(SO_4_) _2_·12H_2_O, 6.42 g MES, 10 ml mineral stock solution [30], and 10 ml vitamin stock solution [30] (pH 6.0). Strains JemMK663^T^ and JemC60 were subcultured in Medium A without NH_4_Cl supplementation, whereas JemR1613^T^ was subcultured in Medium A supplemented with 5 mM NH_4_Cl. JemC56^T^ and JemC63^T^ were subcultured in Medium B with 5 mM NH_4_Cl. For JemR1613^T^, JemC56^T^, and JemC63^T^, growth was severely impaired when NH_4_Cl concentrations were further reduced, and therefore 5 mM NH_4_Cl was required to obtain sufficient biomass for subsequent assays. All strains were incubated at 35°C for 9 d without shaking under anoxic conditions (N_2_/CO_2_, 80:20, v/v). Following this subculture step, cells were used for subsequent *in vitro* nitrogen fixation assays and soil microcosm experiments.

### *In vitro* assessment of nitrogen fixation ability

Cells obtained from the subculture described above were inoculated into 50-ml serum bottles containing 20 ml of the corresponding Medium A or B to an initial OD_600_ of 0.02 and incubated under anoxic conditions (N_2_/CO_2_, 80:20, v/v) at 35°C without shaking. Growth was assessed by measuring the optical density at 600 nm (OD_600_) using a spectrophotometer (ASUV-1100, AS ONE, Osaka, Japan). For the growth curve of strain JemMK663^T^, the OD_600_ was measured daily during an 11 d incubation period. As a control, the same setup was used with pure Ar instead of the N_2_/CO_2_ gas mixture.

The nitrogen fixation activity of the isolated strains, JemMK663^T^, JemC60, JemR1613^T^, and JemC56^T^, was evaluated *in vitro* using a conventional acetylene reduction assay, which quantifies nitrogenase activity by measuring the reduction of acetylene to ethylene [46]. After cultivation under anoxic conditions in Medium A or B for 5 d from an initial OD_600_ of 0.02 as described above, the headspace of the vials was replaced with Ar/acetylene gas (90:10, v/v) and the vials were incubated for an additional 2 d at 35°C. Ethylene production in the headspace was analyzed using a Shimadzu GC-2014 gas chromatograph (Shimadzu Corporation, Kyoto, Japan) equipped with a packed column (Porapak N, 80/100 mesh, 3 mm internal diameter × 1 m length) and a flame ionization detector. Helium was used as the carrier gas at a pressure of 24.5 kPa, with the oven temperature maintained at 70°C. The protein content of the cultured cells was measured using the Qubit Protein Assay Kit (Thermo Fisher Scientific, Waltham, MA, USA) following protein extraction with B-PER Bacterial Protein Extraction Reagent (Thermo Fisher Scientific). A negative control without cells was prepared. The inhibitory effect of ammonium on nitrogen fixation activity in JemMK663^T^ was assessed in Medium A supplemented with a range of NH_4_Cl (0.05, 0.2, 1, and 10 mM).

The following analyses were performed on strain JemMK663^T^, selected based on its phylogenetic relatedness and acetylene reduction activity observed *in vitro*. Similarly, after 5 d of cultivation, RNA was extracted from cells of strain JemMK663^T^ using NucleoSpin RNA Plus (Macherey-Nagel, Düren, Germany), and cDNA was synthesized using ReverTra Ace qPCR RT Master Mix with gDNA Remover (Takara Bio, Shiga, Japan). Quantitative PCR (qPCR) was performed using THUNDERBIRD SYBR qPCR Mix (Toyobo, Osaka, Japan) on a LightCycler 96 (Roche, Basel, Switzerland) using primer sets specific to *nifD* and *rpoB* (Table S2). The transcription levels of both genes were quantified. The thermal cycling was performed as follows: preheat at 95°C for 30 s; 45 cycles of 95°C for 15 s, 60°C for 30 s, and 72°C for 15 s.

To directly evaluate N_2_ uptake, the strain JemMK663^T^ was cultured in Medium A under an Ar/^15^N_2_ atmosphere (75:25, v/v) at 35°C for 10 d in triplicate. A control culture was prepared under identical conditions using ^14^N_2_. Following incubation, cells from 250 ml of the culture per replicate (*n* = 3) were harvested by centrifugation, washed with sterile water, and freeze-dried using an EYELA FDU-2100 freeze dryer (Tokyo Rikakikai, Tokyo, Japan). The ^15^N/^14^N isotope ratio (atom %) was determined using a DELTA Q isotope ratio mass spectrometer (Thermo Fisher Scientific).

### Assessment of nitrogen fixation activity in a soil microcosm

To evaluate nitrogen fixation activity of four *Acidobacteriota* isolates (JemMK663^T^, JemR1613^T^, JemC56^T^, and JemC63^T^) under soil conditions, a microcosm assay using paddy soil from Nagaoka, Japan, was performed following a method previously applied to diazotrophic strains [28]. A soil suspension was prepared by mixing soil and water at 1:3 (w/v) ratio and transferred into 5 ml serum bottles, which were then sealed with butyl rubber stoppers and aluminum crimps. The suspension was preincubated at 30°C without shaking for one week to deplete pre-existing ammonium. After preincubation, the vials were autoclaved and the headspace was replaced with an N_2_/CO_2_ mixture (80:20, v/v). The pH of the soil slurry was 5.8. The *Acidobacteriota* strains were pre-cultured under the following conditions: JemMK663^T^ was cultured in Medium A at 35°C for 2 d; JemR1613^T^ was cultured in Medium A supplemented with 5 mM NH_4_Cl at 35°C for 9 d; JemC56^T^ and JemC63^T^ were cultured in Medium B supplemented with 5 mM NH_4_Cl at 35°C for 9 d. The duration of pre-culture was adjusted for each strain to reach a comparable cell density (OD_600_ in the range of 0.04–0.06) prior to inoculation. For inoculation, 7 ml of each culture was centrifuged at 13 000 rpm, and the harvested cells were washed with oxygen-free sterile water and resuspended in soil microcosms supplemented with 25 mM glucose. The inoculated microcosms were incubated at 35°C for 4 d, after which the headspace was replaced with an Ar/acetylene mixture (90:10, v/v). Following an additional 2-d incubation period, ethylene production in the headspace was measured by gas chromatography as described above, including in the presence of 10 mM NH_4_Cl to assess the inhibitory effect of ammonium. Simultaneously, soil RNA was extracted using the RNeasy PowerSoil Total RNA Kit (Qiagen) and RNA Clean & Concentrator-5 (Zymo Research, CA, USA). cDNA synthesis and qPCR analyses of *nifD* and *rpoB* were performed as described above. The uptake of ^15^N by the inoculated soil was measured by incubating the soil under conditions similar to those described above. The headspace of the soil microcosm was replaced with Ar/^15^N_2_ gas (75:25, v/v) and incubated at 35°C for 10 d. After incubation, the soil was freeze-dried and analyzed for the ratio of ^15^N/^14^N (atom %) using a DELTA Q isotope ratio mass spectrometer (Thermo Fisher Scientific), as described in “*In vitro* assessment of nitrogen fixation ability” section.

### Statistical analysis

All statistical analyses were conducted in R ver. 4.4.2. Differences in acetylene reduction activity under varying NH_4_^+^ concentrations were evaluated using one-way analysis of variance (ANOVA), followed by Dunnett’s multiple comparison test, with the NH_4_^+^-free condition (0 mM) used as the control. Pairwise comparisons of nitrogenase activities (^15^N atom % in cell biomass and acetylene reduction activity under soil conditions) and gene transcriptional activities were conducted using Welch’s two-sample *t-*test. Differences in ^15^N atom % fixed in soil among treatments were assessed using the Dunnett-type nonparametric multiple comparison test implemented in the *nparcomp* package in R, using the “No cell” treatment as the control. One-sided alternative hypotheses (treatment > control) were applied to determine whether bacterial inoculation significantly increased ^15^N_2_ fixation relative to the control.

### Identification of nitrogenase sequences on prokaryotic genomes and inference of their phylogeny

To investigate the distribution of nitrogenase genes among *Acidobacteriota*, three genome datasets, including all representative genomes from GTDB v226 (*n* = 143,614) [42] and a MAG catalog generated from soil metagenomes (*n* = 40,350), were analyzed [47] (see supplementary method for detail). The CDSs were predicted for each genome using Prodigal v2.6.3 with default parameters, followed by the identification of nitrogenase genes using hidden Markov models from TIGRFAM and KOfam (Table S3). To establish consistency in taxonomic notations, genomes in the soil MAG catalog were taxonomically annotated using GTDB-Tk 2.4.1 [48]. Thus obtained NifD (K02586 in KEGG orthology) sequences were aligned using MAFFT v7.525 with “—auto” option [49], and an approximate ML tree was constructed using FastTree v2.1.11 [50]. The same procedure was performed for the NifK (K02591 in KEGG orthology) sequences. We further classified the nitrogenase sequences annotated as *Acidobacteriota* into six or seven distinct groups. The NifD and NifK sequences were used as references to annotate the metagenomic reads, as described in Supplementary Information.

### Metagenomic analyses

Shotgun metagenomic data collected worldwide were reanalyzed to investigate the global distribution of *Acidobacteriota* with nitrogen-fixing potential. We used intermediate files from a previous meta-analysis report (https://plus.figshare.com/articles/dataset/Quality-filtered_soil_shotgun_metagenomes/25332547) [29], which consisted of quality-filtered metagenomic sequences from 1451 samples. We detected nitrogenase and 16S rRNA gene sequences from these datasets and assigned taxonomic annotations to each sequence. In addition, metatranscriptomic data from tundra soils [10], which are limited in geographic scope but sufficiently extensive in sequencing depth, were analyzed to provide supplementary evidence for *in situ* expression of nitrogenase genes. Full details are provided in the Supplementary Information.

## Results and discussion

### Isolation and phenotypic characterization of *Acidobacteriota* strains

Five strains, designated JemMK663^T^, JemC60, JemR1613^T^, JemC56^T^, and JemC63^T^, were isolated from soil samples collected from a grassland, paddy fields, and an upland farm (Table S1). Partial 16S rRNA gene sequences (1,381–1,398 bp) of these strains showed the highest similarity to members of the order *Terriglobales* within the phylum *Acidobacteriota*. Strains JemMK663^T^ and JemC60 exhibited 96.2% similarity to *Alloacidobacterium dinghuense* 4Y35^T^, whereas strains JemC56^T^, JemC63^T^, and JemR1613^T^ showed 94.4%–95.5% similarity to *Candidatus* Koribacter versatilis Ellin345.

**Figure 1:**
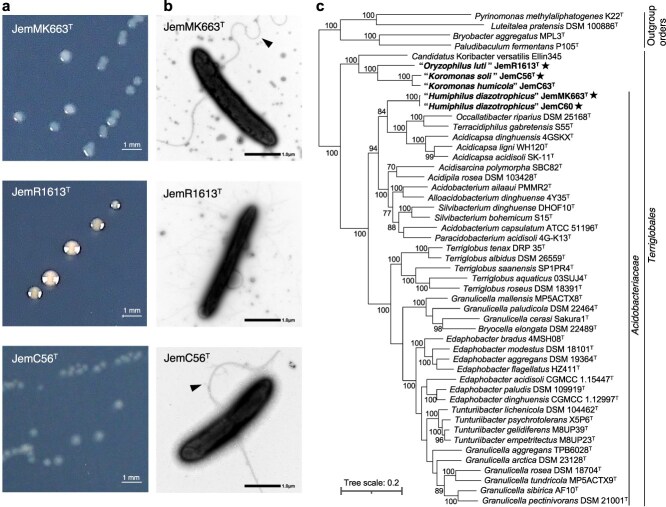
Morphology and phylogenetic placement of the *Acidobacteriota* strains isolated in this study. (a) Colony morphology on R2Af agar plates after 7 d of incubation at 35°C. (b) Transmission electron micrograph showing a single cell. Triangles indicate flagella. (c) Maximum-likelihood phylogenetic tree based on whole-genome sequences, illustrating the position of the five isolates within the *Acidobacteriota* lineage. Isolates obtained in this study are shown in bold. Stars indicate *nif*-harboring strains. Bootstrap values (>70%) are indicated at branch nodes. Colony photographs and TEM images of strains JemC60 and JemC63^T^ are provided in Fig. S1.

Although most of the known members of the order *Terriglobales* are aerobic or microaerophilic, all five strains isolated in this study were facultative anaerobes. Strains JemMK663^T^, JemC60, and JemR1613^T^ grew on R2Af agar plates under both anoxic and oxic conditions, whereas strains JemC56^T^ and JemC63^T^ grew under anoxic or microoxic conditions but did not grow under oxic conditions (Table S1). When cultured under anoxic conditions with glucose as the sole carbon source, all five strains produced propionate as the major metabolic product, suggesting fermentative metabolism (Table S1). Strain JemR1613^T^ formed reddish-orange colonies on R2Af agar plates under anoxic conditions, whereas the other four strains formed whitish-to-beige colonies (Fig. 1a and Fig. S1a). Morphological observations revealed that all five isolates were Gram-stain-negative, non-spore-forming, and rod-shaped. Transmission electron microscopy revealed flagella-like structures in all four strains (excluding JemR1613^T^) (Fig. 1b and Fig. S1b); however, motility was only observed in JemMK663^T^ and JemC60 during microscopic examination in liquid culture (Table S1). All five strains grew at temperatures ranging from 15°C to 45°C (optimum: 35°C–40°C) and at pH values between 4.5 and 8.0 (optimum: 5.5–6.0), and tolerated NaCl concentrations of 0%–0.8% (optimum: 0%–0.2%). The optimal temperature and pH for these isolates were slightly higher than those reported for known members of the family *Acidobacteriaceae* (Table S1) [22]. The native soil pH of the isolation sources ranged from 5.6 to 6.3 (Table S1), which may partly explain the observed pH preference of these isolates. The major fatty acids were iso-C_15:0_ (22.5%–60.1%) and C_16:0_ (19.1%–37.9%) (Table S1). Menaquinone-8 (MK-8) was identified as the predominant respiratory quinone in all five strains, as reported for many known type strains of *Acidobacteriota* members (Table S1) [27, 51–53]. Additional phenotypic traits, including carbon source utilization, enzymatic activities, and antibiotic resistance, are summarized in Table S1.

### Phylogenetic analysis and taxonomic implications

Whole-genome sequencing of the five isolates revealed genome sizes of 3.9–9.2 Mbp with GC contents of 51.5%–61.8%, containing 3,332–7,164 protein-coding genes (Table S4). All strains contained one to four copies of genes encoding cytochrome oxidases for oxygen respiration (Table S5), which is consistent with previous reports on facultative anaerobic *Acidobacteriota* strains [27]. In addition to the high-efficiency *aa*_3_-type cytochrome c oxidase, which serves as the primary terminal oxidase for aerobic respiration under normal oxygen levels, all strains also possessed cytochrome *bd*_1_ quinol oxidase (*cydAB*), which is associated with microaerobic respiration and tolerance to trace oxygen. Regarding dissimilatory nitrogen transformations (excluding nitrogen fixation), none of the strains carried genes encoding dissimilatory nitrate reductase (*narGHI* or *napAB*). The gene set *nrfAH*, which encodes functional proteins for dissimilatory nitrate reduction to ammonia (DNRA), was present only in strains JemC56^T^ and JemC63^T^, whereas strain JemR1613^T^ contained *nrfA* alone (Table S5). Furthermore, none of the strains possessed key denitrification genes *nirK*/*S* or *nosZ*, which encode nitrite reductase or nitrous oxide reductase, respectively.

The pairwise ANI and dDDH values between the five strains and type strains of the order *Terriglobales* ranged from 65.3%–69.0% and 17.9%–31.4%, respectively (Table S6). These values fell below the established thresholds for species delineation (ANI: 95%–96%; dDDH: 60%–70%), indicating that the isolated strains represent distinct species separate from known type strains. Among the isolates, strains JemMK663^T^ and JemC60 shared ANI and dDDH values of 97.6% and 82.7%, respectively, suggesting that they belong to the same species, which was further supported by their identical 16S rRNA gene sequences (100% similarity) and highly similar physiological and chemotaxonomic profiles (Table S1). The AAI values between the isolates and type strains were all 59.7% or less (Table S7), which is below the genus demarcation threshold (70%–76%) proposed in previous studies [30, 32, 54–56]. Strains JemC56^T^ and JemC63^T^ shared 78.4% AAI (Table S7), suggesting that they belong to the same novel genus. However, their ANI and dDDH values (78.7% and 22.2%, respectively) were below the species level thresholds, indicating that they likely represent distinct species within the same genus. Furthermore, strains JemC56^T^, JemC63^T^, and JemR1613^T^ exhibited 49.1%–51.3% AAI against strains JemMK663^T^ and JemC60 and type species within the family *Acidobacteriaceae*. Phylogenomic analysis based on 120 essential single-copy genes revealed that strains JemMK663^T^ and JemC60 clustered with type strains of the family *Acidobacteriaceae*, whereas strains JemC56^T^, JemC63^T^, and JemR1613^T^ formed a distinct clade with *Ca*. Koribacter versatilis Ellin345 (Fig. 1c). This topology was also supported by analyses of the full-length 16S rRNA gene sequences (Fig. S2), although some discrepancies were observed, particularly in the placement of strains JemMK663^T^ and JemC60. *Ca*. Koribacter versatilis Ellin345, isolated from pasture soil under oxic conditions [6, 57], is currently assigned to a putative family (“family 5”) within the order *Terriglobales* according to the latest phylogenetic framework of *Acidobacteriota* [58]. This family is tentatively referred to as “*Candidatus* Korobacteraceae” in the List of Prokaryotic names with Standing in Nomenclature (LPSN) (https://lpsn.dsmz.de/family/korobacteraceae) or “*Koribacteraceae*” in public databases [59, 60]. Taken together, the phylogenomic data support the classification of strains JemMK663^T^ and JemC60 as a novel genus within the family *Acidobacteriaceae*. Strains JemC56^T^ and JemC63^T^ comprise one novel genus, and JemR1613^T^ represents another novel genus; both genera belong to a novel family within the order *Terriglobales*. *Ca*. Koribacter versatilis formed a distinct cluster with strains JemC56^T^, JemC63^T^, and JemR1613^T^ in both 16S rRNA gene-based and phylogenomic analyses (Fig. 1c and Fig. S2), and showed AAI values to these three isolates (53.6%–54.4%, Table S7) within the range reported for members of the family *Acidobacteriaceae* (52.3%–63.6%, Table S7), suggesting that it is likely affiliated with the same family level lineage as these three isolates.

### Nitrogenase gene cluster in *Acidobacteriota* isolates

**Figure 2:**
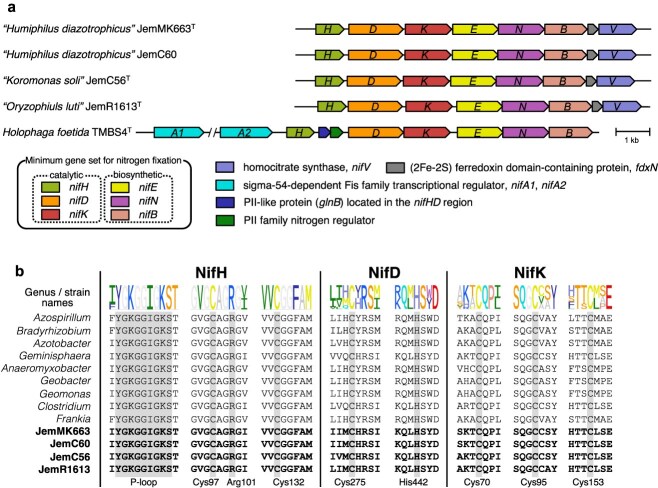
Nitrogenase-coding genes in the isolated *Acidobacteriota* strains. (a) Organization of the *nif* gene cluster in the cultured *Acidobacteriota* strains, including the isolates and *H. foetida* TMBS4^T^. (b) Alignments of conserved regions in NifH, NifD, and NifK. Shaded amino acid residues indicate the MgATP-binding motif (P-loop) and [4Fe–4S] cluster-ligating cysteines in NifH, FeMo cofactor ligands in NifD, and P-cluster ligands in NifK. Numbering of crucial residues follows *Azotobacter vinelandii*, a model diazotroph. Sequence logos are also provided above the multiple sequence alignments.

Among the five isolates, strains JemMK663^T^, JemC60, JemR1613^T^, and JemC56^T^ each possessed a single nitrogenase gene cluster, including the core structural components *nifHDK* and the Fe–Mo cofactor synthesis gene *nifENB* (Fig. 2a). Genes encoding accessory proteins such as *fdxN* and *nifV* were also identified (Fig. 2a). The cluster structure was conserved among the four *Terriglobales* isolates, although this arrangement differed slightly from that observed in *H. foetida* TMBS4^T^ of the *Holophagales* (Fig. 2a). The *nifHDK* sequences of these isolates retained the conserved motifs and ligand-binding residues characteristic of known diazotrophs (Fig. 2b and Table S8), suggesting that the proteins encoded by the *nif* gene cluster in *Acidobacteriota* constitute an active nitrogenase complex.

### Diazotrophic activity of *Acidobacteriota* isolates

**Figure 3:**
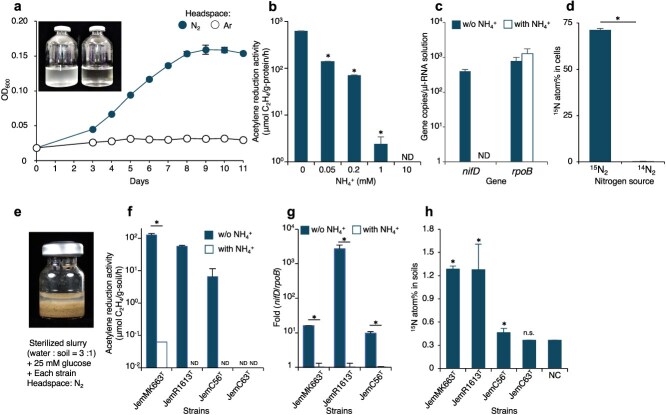
Evaluation of nitrogen fixation activity in the isolated *Acidobacteriota* strains. Panels a–d show assays performed in liquid culture with strain JemMK663^T^. (a) Growth curves in nitrogen-free medium with N_2_ gas as the sole nitrogen source. Inset shows culture appearance after 7 d, with vials sealed with N_2_ gas (left) and Ar gas (right). (b) Acetylene reduction activity, determined by quantifying ethylene production as an indicator of nitrogenase activity under varying NH_4_^+^ concentrations. Significant differences from the NH_4_^+^-free control were detected at all tested concentrations (one-way ANOVA followed by Dunnett’s test, ^*^P <0.05). (c) Transcriptional activity of *nif* genes under nitrogen-fixing conditions. (d) ^15^N incorporation into cellular biomass (atom %). Panels e–h show assays in soil microcosms with strains JemMK663^T^, JemC56^T^, JemR1613^T^, and JemC63^T^. (e) Photograph of soil vials after incubation. (f) Acetylene reduction activity in soil microcosms. (g) Transcriptional activity of *nif* genes in soil. Asterisks in panels d, f, and g indicate significant differences between the relevant groups as determined by Welch’s two-sample *t-*test. ^*^P <0.05. (h) ^15^N incorporation into soils (atom %). Error bars indicate mean ± SE where applicable. Asterisks indicate significant differences compared with the control (NC, “No cell”) as determined by a Dunnett-type nonparametric multiple comparison test (one-sided, treatment > control). ^*^P <0.05; n.s., not significant. Symbols in panel a and bars in panels b, c, d, f, g, and h represent means ± SD (*n* = 3 biological replicates).

When cultured under anoxic conditions in minimal medium containing glucose as the sole carbon source and N_2_ as the sole nitrogen source, strain JemMK663^T^ exhibited N_2_-dependent growth (Fig. 3a). Acetylene reduction activity (6.2 ± 0.8 × 10^2^ μmol C_2_H_4_/g protein/h) was detected in strain JemMK663^T^, and this activity was inhibited by ammonium, with a progressive decrease in activity observed as ammonium concentration increased, and with suppression evident even at micromolar concentrations (0.05 and 0.2 mM) (Fig. 3b). Strain JemC60, a closely related isolate (97.6% ANI to strain JemMK663^T^), also exhibited acetylene reduction activity under the same conditions without ammonium addition (2.1 ± 0.2 × 10^2^ μmol C_2_H_4_/g protein/h). Furthermore, the transcriptional activation of *nifD* was observed under ammonium-depleted conditions (Fig. 3c), and cultivation with ^15^N_2_ as the sole nitrogen source led to a marked increase in ^15^N atom % in the biomass (Fig. 3d), confirming active nitrogen fixation *in vitro*. In contrast, strains JemC56^T^ and JemR1613^T^, both of which possess a nitrogenase gene cluster similar to that of strain JemMK663^T^ (Fig. 2a), did not show N_2_-dependent growth or acetylene reduction activity under the same minimal medium conditions. This suggests that the defined laboratory conditions used here were not suitable for the expression of nitrogenase activity in strains JemC56^T^ and JemR1613^T^. However, when cultured in the soil slurry (Fig. 3e), both strains JemC56^T^ and JemR1613^T^ exhibited measurable acetylene reduction activity comparable to that of strain JemMK663^T^, which was inhibited by ammonium (Fig. 3f). Transcriptional activation of *nifD* in the soil was also detected under ammonium-depleted conditions (Fig. 3g), supporting the regulation of nitrogenase gene expression in response to nitrogen availability. In ^15^N_2_ microcosm experiments, soils inoculated with strains JemMK663^T^, JemR1613^T^, or JemC56^T^ showed a significant increase in ^15^N atom % compared to the uninoculated control (Fig. 3h). In contrast, soils inoculated with strain JemC63^T^, which shares high 16S rRNA gene sequences and AAI similarities with JemC56^T^ (99.4% and 78.7%, respectively) but lacks the *nif* gene cluster, showed neither acetylene reduction activity nor an increase in soil ^15^N atom % (Fig. 3f and h). Collectively, these results indicate that *nif*-harboring *Acidobacteriota* strains exhibit nitrogen fixation activity in soil microcosms. Furthermore, their nitrogenase expression and enzymatic function are regulated in response to nitrogen availability.

### Phylogeny of acidobacterial *nif* genes

**Figure 4:**
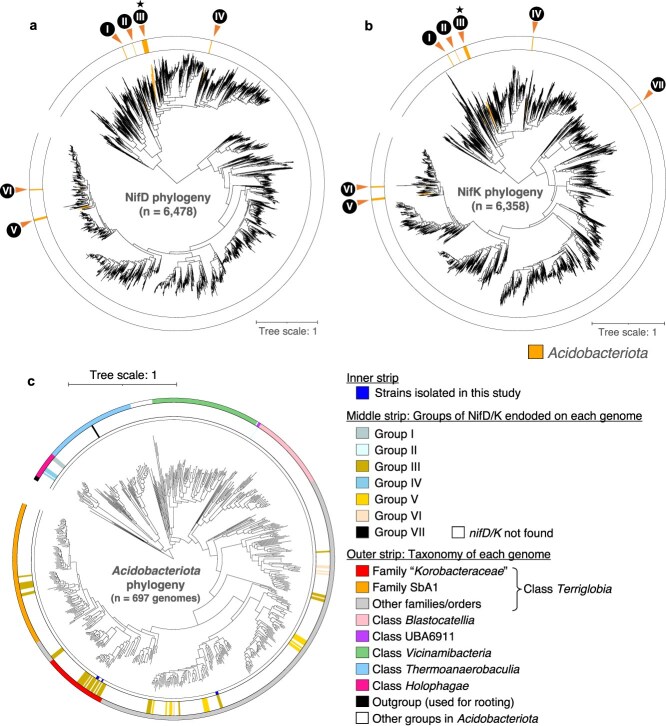
Phylogenetic diversity of *nif*-harboring *Acidobacteriota*. (a) Phylogenetic distribution of acidobacterial NifD among prokaryotic NifD. *Acidobacteriota* NifD are highlighted in orange stripes, forming six distinct groups named Groups I–VI. The group IDs are indicated in closed circles. (b) Phylogenetic distribution of acidobacterial NifK among prokaryotic NifK. NifK of *Acidobacteriota* are highlighted in orange stripes, forming seven distinct groups named Groups I–VII. The group IDs are indicated in closed circles. NifD and NifK belonging to the same group ID never co-exist on a single genome with one exception (see main text). Stars indicate that the four *nif*-harboring strains isolated in this study clustered within Group III in both NifD and NifK phylogenies. (c) Phylogeny of *Acidobacteriota* genomes and distribution of *nif* genes therein. Inner strip highlights five isolates obtained in this study. Middle stripe indicates the groups of NifD/K, as defined in panels (a) and (b), encoded on each genome. Genomes lacking NifD and NifK are not colored. Genomes lacking NifD/K are complete or near-complete (see main text). Outer stripes indicate the family/order/class-level taxonomy of each genome. Taxonomic names presented here are based on GTDB R226, rather than those of ICNP.

We screened more than 190 000 prokaryotic genomes, including MAGs, single cell amplified genomes, and genomes from cultured strains, for nitrogenase sequences, namely NifD and NifK, and analyzed their phylogenetic relationships. Our analysis revealed that NifD/K sequences are prevalent across diverse lineages of *Acidobacteriota* and that their evolutionary history is not straightforward. Seven distinct clades of acidobacterial NifD/K sequences, designated Groups I–VII, were identified (Fig. 4a and b). Each acidobacterial genome encoded only one group of NifD/K, with the exception of MFD04752_bin_1_33, which encoded both Group VI NifD/K and Group III NifD. The phylogeny of NifD/K was loosely congruent with the overall phylogeny of *Acidobacteriota* (Fig. 4c). For example, Groups I and IV NifD/K were derived predominantly from MAGs affiliated with the family *Holophagaceae*. Group V NifD/K consisted solely of MAGs affiliated with the family *Acidobacteriaceae*, whereas Group III NifD/K encompassed multiple families, including “*Korobacteraceae*”, SbA1, and other families/orders within the class *Terriglobia* (Fig. 4c). All *nif*-harboring strains isolated in this study (JemMK663^T^, JemC60, JemC56^T^, and JemR1613^T^) also owned Group III NifD/K (Fig. S3). These findings suggest that nitrogen fixation in *Acidobacteriota* did not originate from a single ancestral event but likely evolved through multiple acquisition, retention, or loss events. In contrast, each of the seven groups of *Acidobacteriota* NifD/K sequences was monophyletic and could be distinguished from the NifD/K sequences of other lineages of prokaryotes.

Among the nitrogenase sequences encoded on our isolated strains, NifD showed highest identity with those of phylum *Bacillota* members, whereas NifH and NifK tend to present higher identity with nitrogenase from *Planctomycetota* or *Verrucomicrobiota* members (Table S9). The evolutionary origin of nitrogenase genes owned by our isolates is not easily identifiable, which calls for further investigation in the future.

### Global occurrence of acidobacterial *nif* genes

**Figure 5:**
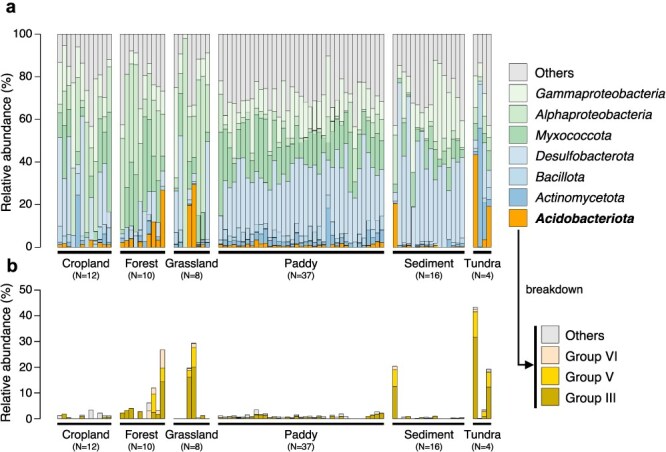
Distribution of *Acidobacteriota nifD*/*nifK* sequences in shotgun metagenomic data from soils. Only datasets with ≥30 reads of *nifD*/*nifK* are presented. (a) Phylum- and *Pseudomonadota* class-level composition of *nifD*/*nifK*. (b) Detailed compositions of *Acidobacteriota nifD*/*nifK* reads. Relative abundances of Groups III, V, and VI are presented. Taxonomic names presented here are based on GTDB R226, rather than those of ICNP.

To assess the environmental prevalence of these acidobacterial *nif* genes, we screened shotgun metagenomic datasets for *nifD/nifK* sequences and assigned their taxonomic affiliations. Among 102 samples where ≥30 reads of *nifD/nifK* were detected, *Acidobacteriota*-derived *nifD* or *nifK* reads were present in 77 samples, with relative abundances ranging from 0.12% to 42.2% (Fig. 5a). They were dominant (>10%) in eight samples from geographically distant locations and diverse land use types (Fig. 5a, Table S10). Further classification of the detected acidobacterial *nifD*/*K* reads revealed that most sequences belonged to Group III, followed by Groups V and VI (Fig. 5b). Similar trends were observed in the metatranscriptomic sequences of the tundra soils (Fig. S4); dominant transcripts of acidobacterial *nif* genes, mainly Groups III and V, were detected in 37 of 45 datasets with ≥30 reads of *nifD*/*nifK* (relative abundances 0.45%–97.0%), indicating that these genes are transcriptionally active in soil environments. Group III not only dominated the environmental datasets but also encompassed all strains isolated in this study that exhibited nitrogen fixation activity (Figs 3 and 4c). This strongly suggests that the most prevalent acidobacterial diazotrophs in soils are functionally active, reinforcing the ecological significance of *Acidobacteriota* as potential contributors to nitrogen fixation in certain environments.

Compared with the ubiquitous dominance of *Acidobacteriota* in soils estimated by rRNA gene-based analyses [3, 29], the prevalence of nitrogen-fixing *Acidobacteriota* appears to be relatively localized. Acidobacterial *nif* genes were abundant (with relative abundances of >10%) in eight samples (Table S10), which were also dominated by 16S rRNA gene reads of *Acidobacteriota* (Fig. S5). Another common feature of these samples, with the exception of oil palm plantation sample, is that they appear to be originated from pristine natural environments such as protected areas or thinly populated regions (Table S10). However, we emphasize that other sites undominated by acidobacterial *nif* are not necessarily affected by extensive human impacts. Tundra samples may be enriched in these genes because of acidic conditions resulting from the incomplete decomposition of plant residues and accumulation of organic acids [61] (Table S10). The most frequently detected sequences belonged to Group III, which includes all strains isolated in this study with an optimum growth at pH 5.5–6.0 (Table S1) and *Acidobacteriaceae* members that generally exhibit optimal growth under mildly acidic conditions (typically pH 4–6) [22]. Future studies should incorporate metagenomic and metatranscriptomic analyses linked with detailed soil physicochemical data to validate these hypotheses.

## Conclusion and outlook

This study provides culture-based experimental evidence of biological nitrogen fixation in the phylum *Acidobacteriota*. By isolating and characterizing multiple strains harboring functional *nif* gene clusters, we demonstrated their ability to fix atmospheric *nitrogen in vitro* and in soil microcosms. These findings suggest that *Acidobacteriota*, previously recognized for their contribution to carbon cycling, play an important role in nitrogen availability in terrestrial ecosystems. Furthermore, global-scale metagenomic analysis showed that *nif*-harboring *Acidobacteriota* are widely distributed across diverse soil environments and are locally dominant in some cases, underscoring their ecological significance. Collectively, this study expands our understanding of the ecological function of *Acidobacteriota* and the soil diazotrophic microbiome, and highlights that *Acidobacteriota*, whose potential for nitrogen fixation has been underestimated, should be considered in analyses of diazotrophic microbiomes.

Among *nif*-harboring *Acidobacteriota*, Group III, which includes members within the class *Terriglobia*, was the most frequently detected in soils and was successfully isolated in this study (Figs 4 and 5b), whereas the other groups remained uncultured. MAG analyses have also frequently detected *nif*-harboring *Acidobacteriota* belonging to the class *Terriglobia* [20, 21], which includes the family *Acidobacteriaceae*, a lineage with relatively many cultured representatives within the phylum *Acidobacteriota* [27, 58, 62]. However, the absence of *nif*-positive isolates in this group has not been reported until now. Isolation in this study was achieved through preincubation using soil slurry without any amendments with C and N growth substrates, suggesting that these nutrient-limited conditions may have favored *Acidobacteriota* adapted to oligotrophic soil environments, including diazotrophic members, although non-diazotrophic strains may also have been recovered due to trace nitrogen sources or potential cross-feeding interactions. Previous isolation efforts for *Acidobacteriota* may have been biased toward oxic conditions, which could have limited the recovery of *nif*-harboring strains adapted to low-oxygen environments. This suggests that combining anoxic or microoxic conditions with enrichment strategies that are both nitrogen-limited and soil-based may facilitate the isolation of additional diazotrophic *Acidobacteriota* lineages that remain uncultured.

Finally, based on integrative taxonomic evidence, including diazotrophic traits, we propose the isolated strains as phylogenetically distinct members of *Acidobacteriota*, comprising one novel family, three novel genera, and four novel species: *Koromonadaceae* fam. nov. (JemC56^T^, JemC63^T^, and JemR1613^T^); *Koromonas soli* gen. nov., sp. nov. (JemC56^T^); *Koromonas humicola* sp. nov. (JemC63^T^); *Oryzophilus luti* gen. nov., sp. nov. (JemR1613^T^); *Humiphilus diazotrophicus* gen. nov., sp. nov. (JemMK663^T^ and JemC60) (Table 1).

**Table 1:** Protologues of one novel family, three novel genera, and four novel species within the class *Terriglobia* of the phylum *Acidobacteriota* proposed in this study.

Proposed name (etymology)	Description
*Humiphilus* gen. nov. (Hu’mi.phi.lus. L. fem. n. *humus*, soil; L. masc. adj. *philus -a -um*, friend, loving; from Gr. adj. *philos* -*ê -on*, loving; N.L. masc. n. *Humiphilus*, a soil-loving bacterium)	Cells are facultatively anaerobic, Gram-stain-negative, non-spore-forming, rod-shaped, and motile by flagella. Cells exhibit fermentative growth under anoxic conditions. Colonies on R2A agar supplemented with fumarate are whitish to beige in color. The predominant quinone is MK-8. The DNA G + C content is 51.5 mol%. The type species is *Humiphilus diazotrophicus*.
*Humiphilus diazotrophicus* sp. nov. (di.a.zo.tro’phi.cus. Gr. adv. *dis*, two, double; N.L. neut. n. *azotum*, nitrogen; Gr. masc. adj. *trophikos*, nursing, tending, or feeding; N.L. masc. adj. *diazotrophicus*, one that feeds on dinitrogen)	Besides the characteristics that define the genus, the following characteristics are observed. Growth occurs at 20°C–40°C (optimum, 35°C), at pH 4.5–8.0 (optimum, 5.5), and with 0%–0.6% (w/v) NaCl (optimum, 0%). Cells are 0.6–0.8 μm wide and 2.5–4.3 μm long. Nitrogen fixation occurs under nitrogen-limited conditions and is inhibited by ammonium, and the genome contains *nif* genes. Glucose, fructose, galactose, mannose, xylose, arabinose, rhamnose, sucrose, maltose, and cellobiose are assimilated, but fucose, gluconate, galacturonate, glucuronate, glycerol, acetate, lactate, succinate, malate, citrate, fumarate, and pyruvate are not. Cells exhibit fermentative growth under anoxic conditions, producing acetate, propionate, and formate as major end products. Xylan and starch are hydrolyzed, but cellulose and pectin are not. Alkaline phosphatase, esterase (C4), esterase lipase (C8), leucine arylamidase, valine arylamidase, cystine arylamidase, trypsin, acid phosphatase, naphthol-AS-BI-phosphohydrolase, α-galactosidase, β-galactosidase, β-glucuronidase, α-glucosidase, β-glucosidase, N-acetyl-β-glucosaminidase, α-mannosidase, and α-fucosidase activities are present, but catalase, oxidase, lipase (C14), and α-chymotrypsin activities are absent. The major components of fatty acids are iso-C_15:0_ and C_16:0_. The genomic DNA G + C content of the type strain is 51.5 mol%. The type strain JemMK663^T^ (=JCM 39634^T^ = KCTC 35016^T^) was isolated from soil in a weed-covered area in Hokkaido, Japan, in 2021. The 16S rRNA gene sequence and whole-genome sequence of strain JemMK663^T^ have been deposited in GenBank/ENA/DDBJ databases under accession numbers PV355031 and AP040866–AP040868, respectively. The species also includes strain C60.
*Koromonadaceae* fam. nov. (Ko.ro.mo.na.da.ce’ae. N.L. fem. n. *Koromonas*, type genus of the family; L. fem. pl. n. suff. *-aceae*, ending to denote a family; N.L. fem. pl. n. *Koromonadaceae*, the *Koromonas* family)	Cells are facultatively anaerobic, Gram-stain-negative, non-spore-forming, and rod-shaped. Cells exhibit fermentative growth under anoxic conditions. The predominant quinone is MK-8. It includes two genera, *Koromonas* and *Oryzophilus*. The DNA G + C content is 55.8–61.8 mol%. The type genus is *Koromonas*.
*Koromonas* gen. nov. (Ko.ro.mo.nas. Gr. fem. n. *Korê*, a name for the Earth goddess Persephone; L. fem. n. *monas*, a unit, monad; N.L. fem. n. *Koromonas*, a monad from the earth)	Besides the characteristics that define the family, the following characteristics are observed. Cells appear non-motile under phase-contrast microscopy despite the presence of flagella and genes encoding flagellar components. Colonies on R2A agar supplemented with fumarate are whitish to beige in color. The DNA G + C content is 55.8–57.2 mol%. The type species is *Koromonas soli*.
*Koromonas soli* sp. nov. (so’li. L. gen. n. *soli*, of soil, the isolation source of the type strain)	Besides the characteristics that define the genus, the following characteristics are observed. Growth occurs at 15°C–40°C (optimum, 35°C), at pH 5.5–6.5 (optimum, 5.5), and with 0%–0.2% (w/v) NaCl (optimum, 0%). Cells are 0.6–0.9 μm wide and 2.9–7.0 μm long. Nitrogen fixation occurs under nitrogen-limited soil conditions and is inhibited by ammonium, and the genome contains *nif* genes. Glucose, mannose, maltose, and cellobiose are assimilated, but fructose, galactose, xylose, arabinose, rhamnose, sucrose, fucose, gluconate, galacturonate, glucuronate, glycerol, acetate, lactate, succinate, malate, citrate, fumarate, and pyruvate are not. Cells exhibit fermentative growth under anoxic conditions, producing propionate and butyric acid as major end products. Starch is hydrolyzed, but cellulose, xylan, and pectin are not. Alkaline phosphatase, esterase (C4), esterase lipase (C8), lipase (C14), leucine arylamidase, valine arylamidase, cystine arylamidase, α-chymotrypsin, acid phosphatase, naphthol-AS-BI-phosphohydrolase, β-galactosidase, β-glucuronidase, α-glucosidase, β-glucosidase activities are present, but catalase, oxidase, trypsin, α-galactosidase, N-acetyl-β-glucosaminidase, α-mannosidase, and α-fucosidase activities are not. The major components of fatty acids are iso-C_15:0_ and C_16:0_. The genomic DNA G + C content of the type strain is 57.2 mol%. The type strain JemC56^T^ (=JCM 39635^T^ = KCTC 35017^T^) was isolated from soil in an upland agricultural field in Hokkaido, Japan, in 2022. The 16S rRNA gene sequence and whole-genome sequence of strain JemC56^T^ have been deposited in GenBank/ENA/DDBJ databases under accession numbers PV355033 and AP040872, respectively.
*Koromonas humicola* sp. nov. (hu.mi’co.la. L. fem. n. *humus*, soil; L. masc./fem. n. suff. *-cola*, inhabiting; N.L. fem. n. *humicola*, soil-inhabiting)	Besides the characteristics that define the genus, the following characteristics are observed. Growth occurs at 30°C–40°C (optimum, 35°C), at pH 5.5–6.5 (optimum, 6.0), and with 0%–0.2% (w/v) NaCl (optimum, 0%). Cells are 0.5–0.7 μm wide and 1.7–4.1 μm long. Nitrogen fixation activity was not detected, and the genome lacks *nif* genes. Glucose, mannose, maltose, and cellobiose are assimilated, but fructose, galactose, xylose, arabinose, rhamnose, sucrose, fucose, gluconate, galacturonate, glucuronate, glycerol, acetate, lactate, succinate, malate, citrate, fumarate, and pyruvate are not. Cells exhibit fermentative growth under anoxic conditions, producing propionate and butyric acid as major end products. Starch is hydrolyzed, but cellulose, xylan, and pectin are not. Alkaline phosphatase, esterase (C4), esterase lipase (C8), leucine arylamidase, valine arylamidase, cystine arylamidase, α-chymotrypsin, acid phosphatase, naphthol-AS-BI-phosphohydrolase, β-glucuronidase, α-glucosidase, β-glucosidase, N-acetyl-β-glucosaminidase, and α-mannosidase activities are present, but catalase, oxidase, lipase (C14), trypsin, α-galactosidase, β-galactosidase, and α-fucosidase activities are not. The major components of fatty acids are iso-C_15:0_ and C_16:0_. The genomic DNA G + C content of the type strain is 55.8 mol%. The type strain JemC63^T^ (=JCM 39636^T^ = KCTC 35018^T^) was isolated from the paddy soil in Okayama, Japan, in 2022. The 16S rRNA gene sequence and whole-genome sequence of strain JemC63^T^ have been deposited in GenBank/ENA/DDBJ databases under accession numbers PV355034 and AP040873, respectively.
*Oryzophilus* gen. nov. (O.ry.zo.phi.lus. Gr. fem. n. *oryza*, rice; L. masc. adj. *philus -a -um*, friend, loving; from Gr. adj. *philos -ê -on*, loving; N.L. masc. n. *Oryzophilus*, a bacterium preferring rice paddy soil)	Besides the characteristics that define the family, the following characteristics are observed. Colonies on R2A agar supplemented with fumarate are reddish-orange in color. The DNA G + C content is 61.8 mol%. The type species is *Oryzophilus luti*.
*Oryzophilus luti* sp. nov. (lu’ti. L. gen. n. *luti*, of mud, the isolation source of the type strain)	Besides the characteristics that define the genus, the following characteristics are observed. Under phase-contrast microscopy, cells appear non-motile despite the presence of genes encoding flagellar components. Growth occurs at 15°C–45°C (optimum, 40°C), at pH 5.5–8.0 (optimum, 6.0), and with 0%–0.8% (w/v) NaCl (optimum, 0%–0.2%). Cells are 0.4 μm wide and 2.2–3.3 μm long. Nitrogen fixation occurs in nitrogen-limited soil conditions and is inhibited by ammonium, and the genome contains *nif* genes. Glucose, mannose, and maltose are assimilated, but fructose, galactose, xylose, arabinose, rhamnose, sucrose, fucose, gluconate, galacturonate, glucuronate, cellobiose, glycerol, acetate, lactate, succinate, malate, citrate, fumarate, and pyruvate are not. Cells exhibit fermentative growth under anoxic conditions, producing propionate as a major end product. Xylan and starch are hydrolyzed, but cellulose and pectin are not. Alkaline phosphatase, esterase (C4), esterase lipase (C8), leucine arylamidase, valine arylamidase, acid phosphatase, naphthol-AS-BI-phosphohydrolase, and β-glucosidase activities are present, but catalase, oxidase, lipase (C14), cystine arylamidase, trypsin, α-chymotrypsin, α-galactosidase, β-galactosidase, β-glucuronidase, α-glucosidase, N-acetyl-β-glucosaminidase, α-mannosidase, and α-fucosidase activities are not. The major components of fatty acids are iso-C_15:0_ and C_16:0_. The genomic DNA G + C content of the type strain is 61.8 mol%. The type strain JemR1613^T^ (=JCM 39637^T^ = KCTC 35019^T^) was isolated from the paddy soil in Chiba, Japan, in 2020. The 16S rRNA gene sequence and whole-genome sequence of strain JemR1613^T^ have been deposited in GenBank/ENA/DDBJ databases under accession numbers PV355035 and AP040874, respectively.

## Supplementary Material

Supplementary_material_wrag157

## Data Availability

Partial 16S rRNA gene sequences and complete genome sequences of five isolates (strains JemMK663^T^, JemC60, Jem56^T^, JemC63^T^, and JemR1613^T^) have been deposited in DDBJ/ENA/GenBank under accession numbers PV355031–PV355035 and AP040866–AP040874, respectively. Bioinformatics scripts and key intermediate files are available at FigShare (https://doi.org/10.6084/m9.figshare.31272766).
