# Supplementary material for: Isolation and global occurrence of nitrogen-fixing Acidobacteriota in soil environments
Source: ISME J. 2026 Jun 18;20(1):wrag157. doi: 10.1093/ismejo/wrag157 (PMC13362970; doi:10.1093/ismejo/wrag157)
Supplement: Supplementary_material_wrag157 [file supplementary_material_wrag157.zip › 04R1_SupInfo260616.pdf]

Supplementary Information

Isolation and global occurrence of nitrogen-fixing *Acidobacteriota* in soil environments

Hideomi Itoh[1*], Kazumori Mise[1,2*], Miyu Kuniyasu[3], Sawa Wasai-Hara[1], Natsumi Ushijima[4]
(*: Equally contributed)

[1]Biomanufacturing Process Research Center, National Institute of Advanced Industrial Science and Technology (AIST) Hokkaido, 2-17-2-1 Tsukisamu-higashi, Toyohira-ku, Sapporo, Hokkaido 062-8517, Japan

[2]Institute of Low Temperature Science, Hokkaido University. Kita 19, Nishi 8, Kita-ku, Sapporo, Hokkaido 060-0819, Japan

[3]Department of Biotechnology, Hokkaido High-technology College, 2-12-1, Megumino-kita, Eniwa, Hokkaido 061-1396, Japan

[4]Graduate School of Dental Medicine, Hokkaido University, Kita 13, Nishi 7, Kita-ku, Sapporo, Hokkaido 060-8586, Japan

Corresponding authors:

Hideomi Itoh (hideomi-itou@aist.go.jp)

Kazumori Mise (mise-33@lowtem.hokudai.ac.jp)

Contents:

Figures S1–S5

Tables S1–S10

Supplementary methods

Preparation of genomic DNA

Genomic DNA was extracted from the cells harvested in R2Af broth using different methods for each strain. Strain JemMK663^{T}: Genomic DNA was extracted using Lysis Solution F (NIPPON GENE, Tokyo, Japan), followed by grinding with a Shake Master Neo (BMS, Tokyo, Japan). After incubation at 65°C for 10 min and centrifugation, the resulting supernatant was purified using the MPure-12 system (MP Bio, Tokyo, Japan), AMPure XP beads (Beckman Coulter, CA, USA), PowerClean Pro Kit (Qiagen, Hilden, Germany), and Short Read Eliminator XS (Circulomics, CA, USA). Strain JemC60: DNA was extracted using a Genomic-tip 20G (Qiagen), followed by purification with Short Read Eliminator XS (Circulomics) and DNA Clean Beads (MGI Tech, Shenzhen, China). Strain JemC56^{T}: Cells were treated with RNase A (NIPPON GENE) and MetaPolyzyme (Sigma-Aldrich, MO, USA) in PBS buffer, followed by protein digestion with Proteinase K (Takara, Shiga, Japan) and purification with a Genomic-tip 20G (Qiagen). Strain JemC63^{T}: Cells were crushed in liquid nitrogen, and DNA was extracted using a Genomic-tip 20G (Qiagen), followed by purification with ProNex Beads (Promega, WI, USA). Strain R1613^{T}: Genomic DNA was prepared using the phenol/chlorohorm/isoamyl alcohol extraction method and ethanol precipitation, as described previously [1]. DNA integrity was evaluated using a Quantus Fluorometer (Promega) and a 5200 Fragment Analyzer System (Agilent Technologies, CA, USA) with an Agilent HS Genomic DNA 50 kb Kit (Agilent Technologies).

Genomic sequencing

DNA sequencing was performed using the PacBio long-read sequencing systems Sequel IIe (for strains JemMK663^{T} and JemC60) or Revio (for strains JemC56^{T} and JemC63^{T}) (Pacific Biosciences, CA, USA) or by combining short- and long-read sequencing technologies using DNBSEQ-G400 (BGI, Shenzhen, China) and GridION X5 (Oxford Nanopore Technologies, Oxford, UK) (for strain R1613^{T}). For the PacBio long-read data, adapter sequences and reads with an average Q score below 20 were removed using SMRT Link ver. 10.1.0 or 12.0.0 and reads shorter than 1,000 bp were filtered out using Filtlong ver 0.2.0.

Qualified reads were assembled using the Flye ver. 2.9 with default parameters. For hybrid assembly of short- and long-read data, adapter sequences from short reads were removed using Cutadapt ver. 2.7. Low-quality bases with scores below 20 were filtered using Sickle ver. 1.33. Reads shorter than 127 bp, along with their paired reads, were discarded. For long reads, the adapter sequences were removed using Porechop ver. 0.2.3, and reads shorter than 1,000 bp were filtered using FiltLong ver. 0.2.0. The final hybrid assembly of the short and long reads was performed using Unicycler ver. 0.4.7 with default settings. The quality of all constructed genomes was assessed using CheckM2 version 1.1.0 with the default parameters [2].

Physiological and chemotaxonomic characterization of isolated *Acidobacteriota* strains

Unless otherwise specified, all the physiological tests were conducted under anoxic conditions. For plate cultures, anoxic conditions were maintained using an AnaeroPack system (Mitsubishi Gas Chemical, Tokyo, Japan), whereas for liquid cultures, the headspace gas was replaced with N_2/CO_2 (80:20, v/v).

Cells grown on R2Af agar plates at 35°C for one week were stained using neo-B&M Wako reagents (FUJIFILM Wako Pure Chemical Co., Osaka, Tokyo) and a Wirtz spore-staining kit (Muto Pure Chemicals Co., Tokyo, Japan) and then observed under a phase-contrast microscope (ECLIPSE E100, Nikon, Tokyo, Japan). For motility assessment, cells cultured in R2Af medium at 35°C without shaking were examined on the 1st, 3rd, and 5th days of incubation using phase-contrast microscopy (ECLIPSE E100, Nikon). The cell morphology and flagella were further analyzed after negative staining with ammonium molybdate using a JEM-1400 transmission electron microscope (TEM, JEOL Co., Tokyo, Japan). Cell size was measured from the TEM images of cells (n = 4) using ImageJ ver. 1.54g.

The temperature range for growth was determined by incubating cultures in R2Af medium at temperatures ranging from 4°C to 45°C (4°C, 10°C, 15°C, 20°C, 25°C, 30°C, 35°C, 40°C, and 45°C) for one week. The optimal pH and pH range for growth were assessed using R2Af medium adjusted to pH values from 3.0 to 10.0 in 0.5-unit increments at 35°C for one week. The pH of the medium was buffered with citrate buffer for pH 3.0–5.5, MES for pH 5.5–6.5, PIPES for pH 6.5–7.0, HEPES for pH 7.5–8.0, Tricine for pH 8.5, TAPS for pH 9.0, and CHES for pH 9.5–10.0, each at a final concentration of 50 mM.

NaCl tolerance was evaluated by supplementing R2Af medium with NaCl concentrations ranging from 0% to 1.0% in 0.2% increments and incubating at 35°C for one week. To assess growth under oxic (approximately 20% O_2) or microoxic (7–8% O_2) conditions, cultures were incubated on R2Af agar at 35°C for one week under standard atmospheric conditions or with the AnaeroPack-MicroAero (Mitsubishi Gas Chemical), respectively.

Carbon source availability was tested in Medium A (for strains JemMK663^T, JemC60, and JemR1613^T) or Medium B (for strains JemC56^T and JemC63^T); the detailed composition of each medium is described in the main text. Each medium was supplemented with the respective carbon source at a final concentration of 25 mM and 10 mM NH_4Cl. Cultures were incubated at 35°C for one week without shaking. For the measurement of fermentative products, strains were cultured under anoxic conditions in Medium A or Medium B supplemented with 25 mM glucose and 10 mM ammonium at 35°C for one week. The cultured supernatants were filtered through 0.22 μm membranes and analyzed by high-performance liquid chromatography using a Nexera Organic Acid Analysis System (Shimadzu, Kyoto, Japan) equipped with three Shim-pack Fast-OA columns (Shimadzu). Polysaccharide hydrolysis activity was assessed by culturing strains on 1.5% agar plates containing Medium A (for strains JemMK663^T, JemC60, and JemR1613^T) or Medium B (for strains JemC56^T and JemC63^T), supplemented with 0.5% (w/v) of each polysaccharide (CM-cellulose, xylan, pectin, and starch) and 10 mM NH_4Cl, at 35°C for one week. After incubation, hydrolysis was evaluated using the following appropriate staining methods: 0.1% Congo Red for CM-cellulose and xylan, 1% cetyltrimethylammonium bromide for pectin, and iodine solution (available iodine, 1 mg/mL) for starch.

Catalase activity was assessed by observing bubble formation after applying 3% hydrogen peroxide to the colonies grown on R2Af agar plates. Oxidase activity was evaluated using API oxidase reagent according to the manufacturer's protocol (bioMérieux, Lyon, France). Additional enzymatic activities were examined with the API ZYM system (bioMérieux) according to the manufacturer's instructions at 35°C for 5 hours. Antibiotic resistance testing was performed on R2A agar supplemented with each of the 12 antibiotics as described previously [3].

Cellular fatty acid methyl esters and isoprenoid quinones were extracted from cells grown on R2Af agar plates at 35°C for one week, according to the protocol in [3]. The fatty acid composition was analyzed using the MIDI Sherlock Microbial Identification System (version 6.0; MIDI). Quinones were purified using Sep-Pak Plus silica cartridges (Waters, USA) and subsequently analyzed by ultra-performance liquid chromatography on an ACQUITY H-Class system (Waters) equipped with a BEH C18 column (Waters).

Custom database of NifD and NifK and their phylogeny

To elucidate the phylogeny and distribution of nitrogenase sequences among prokaryotes, with a focus on *Acidobacteriota*, we screened the following three bundles of genomes: all representative genomes of GTDB226 (n = 143,614) [4], the SMAG catalogue (n = 40,350) [5], and a MAG catalogue of Danish terrestrial environments from the Microflora Danica project (n = 15,638) [6]. The GTDB provides an extensive overview of the known diversity of prokaryotic genomes, whereas the other two datasets include tens of thousands of soil MAGs. The Microflora Danica dataset relies on deep, long-read sequencing data and includes high-quality MAGs that are difficult to obtain from complex soil microbiomes. The same procedure was applied to the five genomes of our isolates (JemC56^T, JemC60, JemC63^T, JemR1613^T, and JemMK663^T), four of which (excluding JemC63^T) harbored nitrogenase genes. To establish consistency in taxonomic notation, we reannotated SMAG, Microflora Danica MAGs, and genomes of our new isolates according to GTDB R226 [4] using GTDB-Tk v2.4.1 [7] ("--classify" command with "skip-skani" option).

For each genome, we used prodigal v2.6.3 [8] (default parameter settings) to predict coding sequences (CDSs). We then searched for NifH/D/K sequences using two sets of HMM profiles, TIGRFAM [9] and KOfam [10] (**Table S3**), and the hmmscan command in HMMER v3.4 [11]. An amino acid sequence was regarded as NifD if and only if it received HMM bitscores above the default thresholds for both TIGRFAM and KOfam profiles; the same was applied to NifK and NifH.

From the sequences annotated as NifD or NifK, we eliminated sequences containing atypical amino acids or "X" ("wild card" of amino acid), or those annotated as more than one of NifH/D/K (possibly

fused genes). The filtered NifD and NifK sequences were aligned using MAFFT v7.525 [12] (default parameters with "--auto" flag) and an approximate maximum likelihood (ML) tree was constructed using FastTree version 2.1.11 [13]. Here, we focused on NifD and NifK, rather than NifH, as markers, because *nifH* sequences are difficult to distinguish from their homologs that are irrelevant for nitrogen fixation [14].

We also mapped the NifHDK sequences of the four isolates (JemC56^T, JemC60, JemR1613^T, and JemMK663^T) onto our custom database using Needleman-Wunsch algorithm implemented in SWORD v1.0.4 (options: -A NW -a 10000) [15] to speculate on their evolutionary origin. Among the hits with low e-values, we manually determined the best hits based on three different criteria: (i) top hit among sequences from acidobacterial genomes (excluding those from our isolates), (ii) top hit among sequences from non-acidobacterial genomes, and (iii) top hit among sequences from taxonomically characterized isolate genomes.

To determine the root of the NifD tree, we constructed another NifD tree using NifE sequences that served as an outgroup. Specifically, all NifD sequences and seed sequences of TIGRFAM TIGR01283.1 (corresponding to NifE) were aligned using MAFFT and an approximate ML tree was constructed using FastTree. Based on the topology of this tree, we predicted the root of the tree, which separates the 6478 NifD sequences into 2002 and 4476 sequences. These two sets of sequences were monophyletic in a tree consisting only of NifD (**Fig. 4A**). The same applies to the NifK tree; its root was determined using the seed sequences of TIGRFAM TIGR01285.1 (corresponding to NifN). The root separated the 6358 NifK sequences into 1781 and 4577 sequences, and the same branching was observed in the NifK-only tree (**Fig. 4B**). Therefore, we rooted the trees accordingly. Newick utilities [16] were used to reroot the trees. Multiple sequence alignment (MSA) of amino acid sequences and rerooted trees were packaged using Taxtastic v0.11.1 for shotgun metagenomic analysis.

Alignment of conserved regions in NifHDK

Certain critical motifs and residues in nitrogenases are conserved across the diverse lineages of known diazotrophs. To confirm that they are also conserved in *Acidobacteriota* nitrogenases, we obtained NifH,

NifD, and NifK sequences of nine diazotrophs, namely *Azospirillum brasilense* Sp7^T, *Bradyrhizobium diazoefficiens* USDA 110^T, *Azotobacter vinelandii* DJ, *Geminisphaera colitermitum* TAV2^T (annotated as *Diplosphaera* in RefSeq), *Anaeromyxobacter diazotrophicus* Red267^T, *Geobacter sulfurreducens* KN400, *Geomonas oryzae* S43^T, *Clostridium acetobutylicum* ATCC 824^T, and *Frankia casuarinae* CcI3^T, from RefSeq (**Table S10**). NifH of these nine strains and the four *Acidobacteriota* isolates obtained in this study were aligned using MAFFT v7.525 (default parameter settings under "--auto" mode); NifD and NifK were also aligned in the same manner. Nine critical motifs and residues were identified from multiple sequence alignments through manual scrutiny. Sequence logos were generated using the ggmsa package in R, version 4.5.2.

Shotgun metagenomic analyses

To investigate the distribution of acidobacterial nitrogenase genes in the soil, we reanalyzed a global-scale collection of soil metagenomic data [17, 18]. The dataset comprises 1451 shotgun metagenomic datasets. In advance of the metagenomic analysis, we supplemented annotations of eggNOG database v5 [19] by screening for NifD and NifK sequences. We performed HMM search in the same way as described above, and a sequence was determined as NifD or NifK it received bit scores above default thresholds for both TIGRFAM and KOfam profiles.

To identify the *nifD*/*K* sequences from the metagenomic sequences, we performed a two-step homology search. First, all reads were mapped onto a small database of NifD/K, which consists of NifD and NifK sequences in the eggNOG database v5 as described above. We used the blastx command in DIAMOND v2.1.11 [20], with the options "-e 1e-5 -k 1", and a read (i.e., a query sequence) was regarded as a candidate for *nifD*/*K* if and only if a significant hit was found in this step. The candidate reads were mapped to the complete eggNOG database. We predicted CDSs from each read using prodigal v2.6.3 with the option "-p meta", and the predicted amino acid sequences of the CDSs were subjected to homology search using the blastp command in DIAMOND with the options "-e 1e-5 -k 200". We retrieved up to 200

hits per query to increase sensitivity and used only the top hit among the ~200 hits to distinguish *nifD*/*K* reads from other reads.

The NifD/K sequences (amino acid sequences) from the metagenomes were annotated using pplacer and guppy. This procedure was performed as previously described [21]. First, each NifD/K sequence was mapped onto the MSA of the NifD/K reference sequences using MAFFT, and its phylogenetic placement was estimated using pplacer [22]. Taxonomic annotation of the metagenomic sequence was then determined using guppy (the confidence threshold value was set at 0.499999 to allow for floating-point errors).

We also analyzed 16S rRNA gene sequences in each metagenomic dataset as described in [23].

Shotgun metatranscriptomic analyses

The shotgun metatranscriptomic sequences of tundra soils (NCBI BioProject PRJNA386568) [24] were retrieved from the NCBI SRA database. Low-quality regions of the raw reads were trimmed using the VSEARCH v2.30.0 "--fastq_filter" command with the option "--fastq_truncee 1" [25]. The trimmed paired-end reads were merged using VSEARCH "--fastq_mergepairs" command with the options "--fastq_allowmergestagger --fastq_maxdiffpct 10". The merged sequences, as well as forward reads of unmerged read pairs, were quality-filtered using VSEARCH "--fastq_filter" command with the option "--fastq_maxee 0.5". Among the filtered sequences, those shorter than 150 bp were excluded. The filtered metatranscriptomic sequences were analyzed using the same procedure as for shotgun metagenomic analysis.

Throughout this study, iTOL [26], SeqKit [27], and TaxonKit [28] were used where needed.

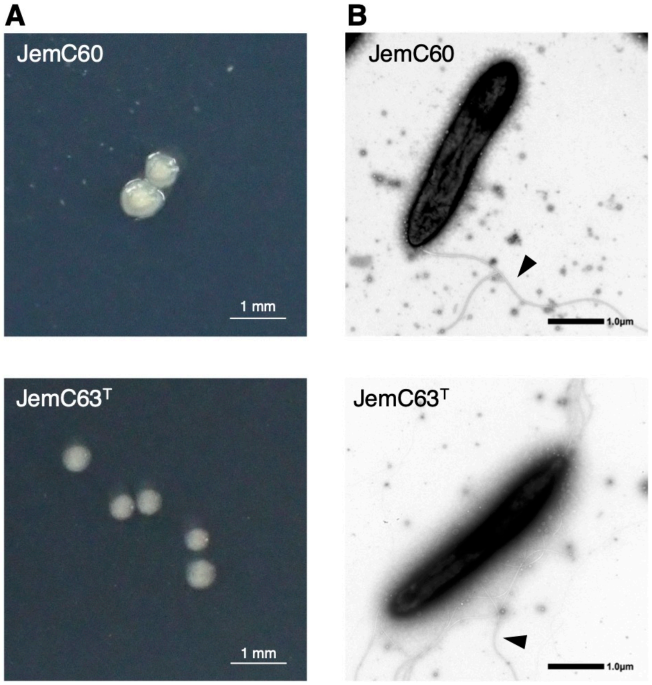

Figure S1. Morphology of the *Acidobacteriota* strains, JemC60 and JemC63^T, isolated in this study. **(A)** Colony morphology on R2Af agar plates after 7 d of incubation at 35°C. **(B)** Transmission electron micrograph showing a single cell. Triangles indicate flagella.

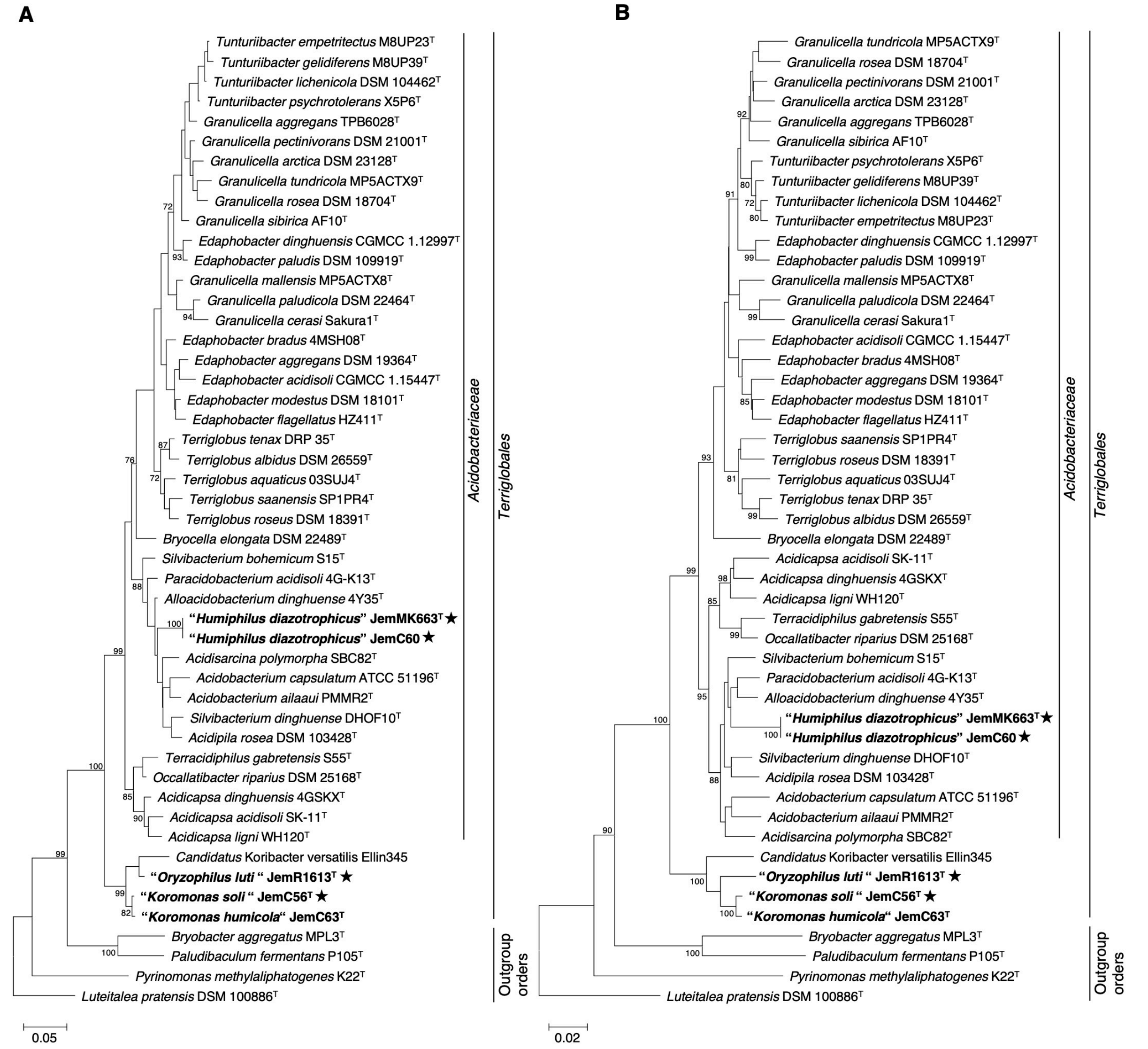

Figure S2. Maximum likelihood (ML, panel **A**) and neighbor-joining (NJ, panel **B**) phylogenetic trees based on full-length 16S rRNA gene sequences retrieved from genomic sequences of *Acidobacteriota* isolates. Both trees were constructed based on the Tamura–Nei model. Bootstrap support values ≥ 70% are indicated at the nodes. Isolates obtained in this study are shown in bold. Stars indicate *nif*-harboring strains.

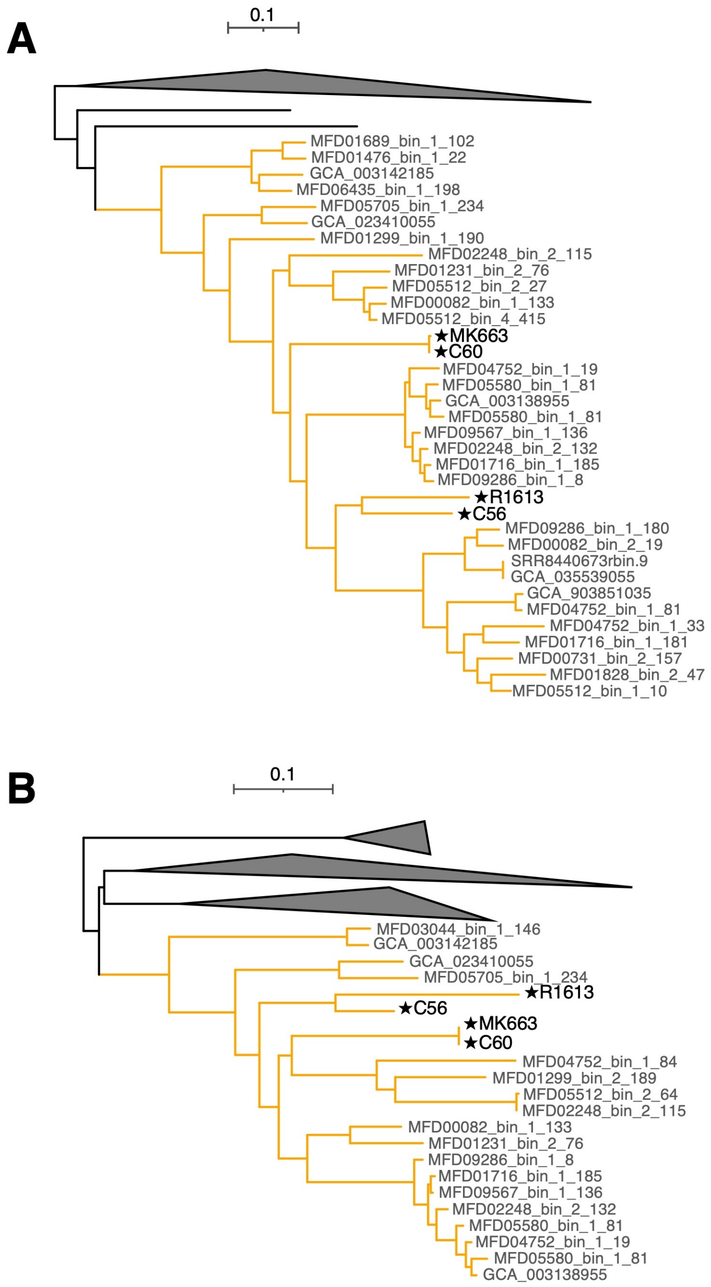

Figure S3. A magnified phylogenetic tree of group III Acidobacteriota NifD (A) and NifK (B). Orange branches indicate group III *Acidobacteriota* NifD/K, whereas black branches and collapsed branches indicate outer groups. Stars indicate NifD/K from four strains that were newly isolated in this study.

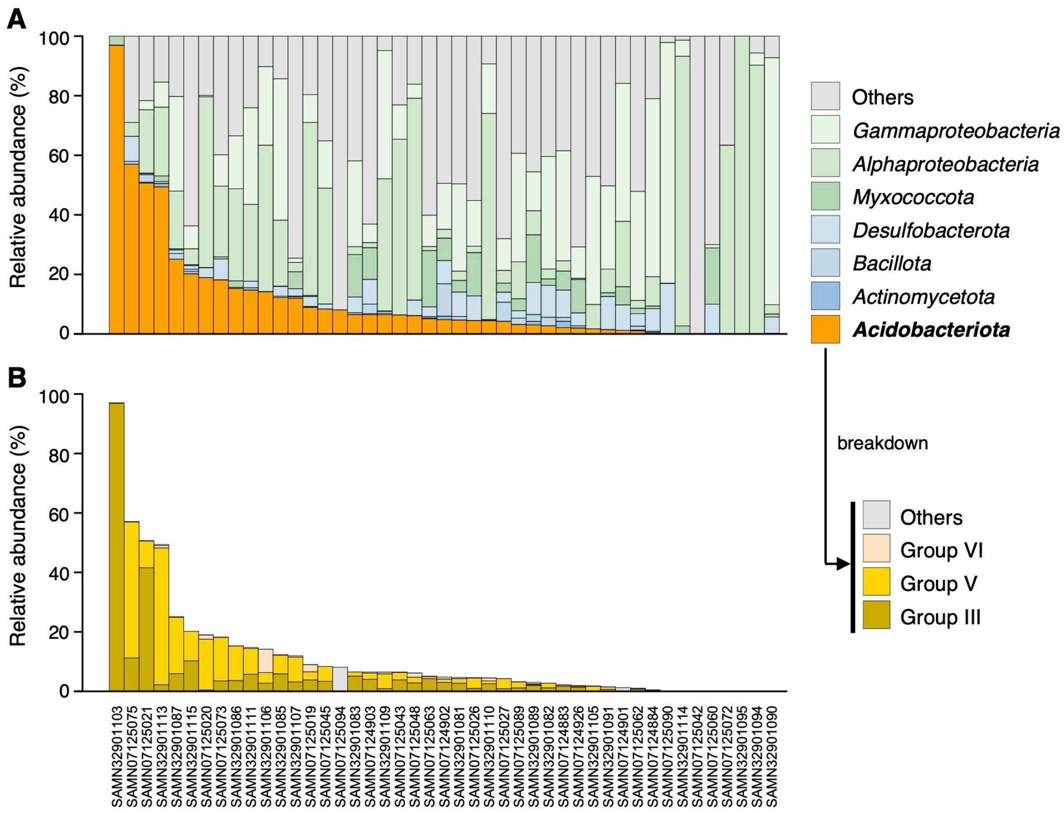

Figure S4. Distribution of *Acidobaceriota nifD*/*nifK* sequences in shotgun metatranscriptomic data from tundra soils, derived from the study by Woodcroft et al. (2018) [24]. Only datasets with ≥ 30 reads of *nifD*/*nifK* are presented. (**A**) Phylum- and *Pseudomonadota* class-level composition of *nifD*/*nifK*. (**B**) Detailed compositions of *Acidobacteriota nifD*/*nifK* reads. Relative abundances of Groups III, V, and VI (defined in **Fig. 4**) are presented. Taxonomic names presented here are based on GTDB R226, rather than those of ICNP.

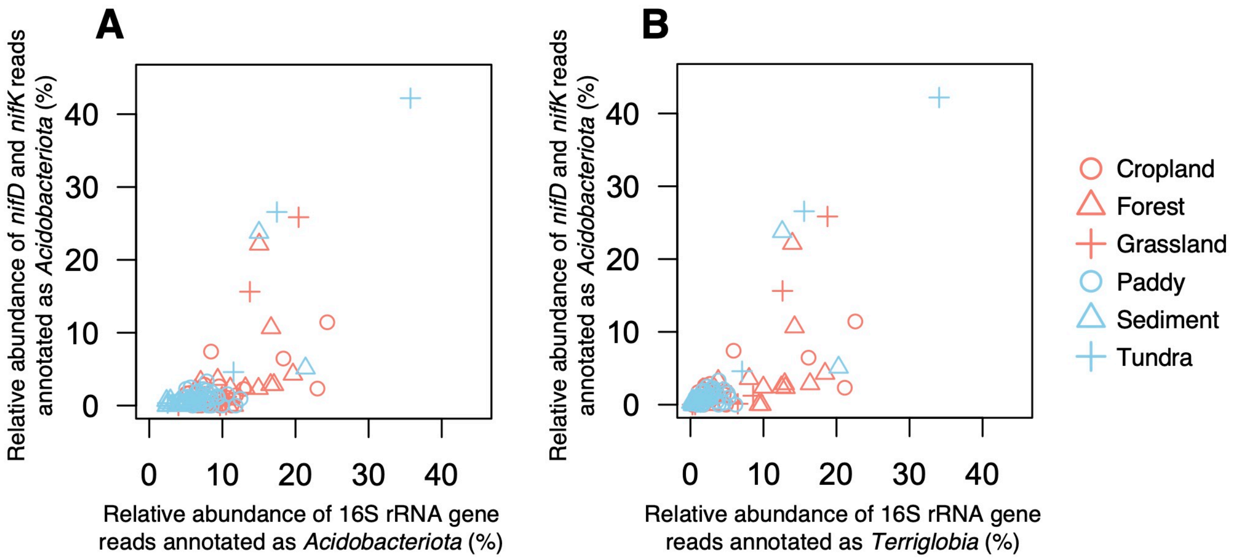

Figure S5. Correlation between taxonomic abundance and *nif* gene abundance in *Acidobacteriota*. (**A**) Correlation between relative abundances of *Acidobacteriota* 16S rRNA gene reads and *Acidobacteriota nif* gene reads. (**B**) Correlation between relative abundances of *Terriglobia* 16S rRNA gene reads and *Acidobacteriota nif* gene reads.

Table S1. Phenotypic and chemotaxonomic characteristics of the five *Acidobacteriota* strains isolated in this study (provided in a separate Excel sheet). [29] [30] [31] [32] [33] [34] [35] [36] [37]

Table S2. Primers used in qPCR analyses.

Name	Strain	Target gene	Direction	Sequence (5'—3')
nifD-F1	JemMK663^T	*nifD*	Forward	CGGACTACCCCGGGAATTAT
nifD-R1			Reverse	AGCCTATCGGGCCATGAGTA
rpoB-F1		*rpoB*	Forward	TAAGCTCTCGGTCGGTGACA
rpoB-R1			Reverse	CACGATCTCGACAGGAGTGC
nifD-Fc56	JemC56^T	*nifD*	Forward	CAATGTGTTCGGGTTCTCCT
nifD-Rc56			Reverse	CGTTCAGTCCAATCACATGC
rpoB-Fc56		*rpoB*	Forward	GCTTCATCGAGTCGCCTTAC
rpoB-Rc56			Reverse	GTGATCGCCCACCTTGTACT
rpoB-Fc63	JemC63^T	*rpoB*	Forward	GGCAAACAATCGCACCTATT
rpoB-Rc63			Reverse	CAGGAAGATCGTTCCCAAAA
nifD-Fr1613	JemR1613^T	*nifD*	Forward	CGAGATGGAAGAAGGACAGC
nifD-Rr1613			Reverse	AATGCCCATCTTCTGTACGC
rpoB-Fr1613		*rpoB*	Forward	CGATGGAGATCGTCCTGAAT
rpoB-Rr1613			Reverse	CCTTGTTCTCCTCGAGCATC

Table S3. A list of HMM profiles used for screening NifH/D/K sequences on prokaryotic genomes.

Source	Family ID	Corresponding ortholog
TIGRFAM	TIGR01282.1	NifD
	TIGR01286.1	NifK
	TIGR01287.1	NifH
KOfam	K02586	NifD
	K02588	NifH
	K02591	NifK

Table S4. Genomic information of the five *Acidobacteriota* strains isolated in this study.

Attribute	Strain				
	JemMK663^{T}	JemC60	JemC56^{T}	JemC63^{T}	JemR1613^{T}
Number of contigs[a]	3	3	1	1	1
Coverage (×)	24.6	27.7	55.7	53.9	663.8
Genome size (b)	9,205,032	9,037,038	4,446,836	4,613,316	3,909,419
Number of protein coding genes	7,164	6,998	3,919	4,061	3,332
DNA G + C content (mol%)	51.5	51.5	57.2	55.8	61.8
Number of rRNAs	6	6	3	6	3
Number of tRNAs	47	48	50	51	50
Number of CRISPRs	3	1	0	0	0
Completeness (%)	99.04	99.04	100.0	99.99	99.96
Contamination (%)	3.99	2.5	0.77	1.52	0.12
Accession Number	AP040866-AP040868	AP040869-AP040871	AP040872	AP040873	AP040874

[a] Strains JemMK663^{T} and JemC60 possess a single circular chromosomal genome and two plasmids.

Table S5. Functional genes encoding cytochrome oxidases and dissimilatory nitrite reductase for DNRA in the five isolated strains.

Strain	Locus ID in the genome		
	aa_3-type cytochrome *c* oxidase (*coxABC*)	cytochrome bd_1 ubiquinol oxidase (*cydAB*)	Nitrite reductase (cytochrome; ammonia-forming) *(nrfAH)*
"Humiphilus diazotrophicus" JemMK663^{T}	ACIDJEMMK663_33040-33060<br>ACIDJEMMK663_65380-65410	ACIDJEMMK663_06980-06990<br>ACIDJEMMK663_13390-13400	-
"Humiphilus diazotrophicus" JemC60	ACIDJEMC60_06510-06530<br>ACIDJEMC60_07540-07560<br>ACIDJEMC60_34780-34810	ACIDJEMC60_25440-25450<br>ACIDJEMC60_66480-66490	-
"Koromonas soli" JemC56^{T}	ACIDJEMC56_23200-23230	ACIDJEMC56_11680-11690	ACIDJEMC56_31570-31580
"Koromonas humicola" JemC63^{T}	ACIDJEMC63_04710-04730<br>ACIDJEMC63_06880-06900<br>ACIDJEMC63_07800-07820<br>ACIDJEMC63_27910-27940	ACIDJEMC63_17030-17040	ACIDJEMC63_38030-38040
"Oryzophilus luti" JemR1613^{T}	ACIDJEMR1613_20580-20600	ACIDJEMR1613_15070-15080	ACIDJEMR1613_21250 (*nrfA*)

Table S6. ANI and dDDH values among the five isolated strains and related strains belonging to the order *Terriglobales* (provided in a separate Excel sheet).

Table S7. Pairwise AAI matrix among the five isolates and related strains belonging to the order *Terriglobales* (provided in a separate Excel sheet). [38]

Table S8. A list of *nifHDK* genes encoded on the newly isolated *Acidobacteriota* strains, their locus tags, corresponding GenBank protein IDs, and summary of their closest homologs based on three different criteria (closest homologs among acidobacterial genomes [excluding our isolates presented in this paper], those among non-acidobacterial genomes, and those among genomes of type strains under International Code of Nomenclature of Prokaryotes [ICNP]). Provided in a separate Excel sheet.

Table S9. A list of metagenomic datasets in which > 10% of *nifD/K* reads were derived from *Acidobacteriota*. Sample IDs and locations are cited from [17, 18].

Environment	Sample ID	Location	Extra information
Cropland	C016	2.05° S, 102.75° E	Oil palm plantation with fertilization
Forest	F179	29.68–29.69° N, 81.96° W	Ordway Swisher Biological Station
	F188	38.90° N, 79.14° W	Smithsonian Conservation Biology Institute
Grassland	G008	28.04–28.05° N, 81.39° W	Disney Wilderness Preserve
	G009	28.07° N, 81.42–81.43° W	Disney Wilderness Preserve
Sediment	S002	42.46° N, 72.23° W	Wetland in Harvard Forest
Tundra	T001	63.88° N, 149.23° W	Soil pH: 4.44 – 5.23
	T004	68.35° N, 19.05° E	

Table S10. A list of nitrogenase sequences used for the analyses of conserved motifs in nitrogenase. Literatures providing experimental evidence for the functionalities of each nitrogenase, such as acetylene-

reducing activity, incorporation of dinitrogen labeled with ^{15}N, or growth in an N-free medium, are also indicated.

**Strain names**	**RefSeq Protein ID**			**Reference providing physiological evidence for nitrogenase functionality**
	**NifH**	**NifD**	**NifK**	
Azospirillum brasilense Sp7^{T}	WP_014239786.1	WP_035672289.1	WP_035672317.1	[39]
Bradyrhizobium diazoefficiens USDA 110^{T}	WP_011084578.1	WP_011084552.1	WP_011084553.1	[40]
Azotobacter vinelandii DJ	WP_012698831.1	WP_012698832.1	WP_012698833.1	[41]
Geminisphaera colitermitum TAV2^{T} (Also noted as "*Diplosphaera*")	WP_043581909.1	WP_081721723.1	WP_043581908.1	[42]
Anaeromyxobacter diazotrophicus Red267^{T}	WP_176067910.1	WP_176067912.1	WP_176067914.1	[43]
Geobacter sulfurreducens KN400	WP_010943447.1	WP_010943446.1	WP_010943445.1	[44]
Geomonas oryzae S43^{T}	WP_129125540.1	WP_129125539.1	WP_129125538.1	[17]
Clostridium acetobutylicum ATCC 824^{T}	WP_010963576.1	WP_010963579.1	WP_010963580.1	[45]
Frankia casuarinae CcI3^{T}	WP_011438842.1	WP_011438841.1	WP_011438840.1	[46]

REFERENCES

1. Itoh H, Kawano K, Kihara M. Draft genome sequence of *Agarivorans* sp. strain Toyoura001, isolated from an abalone gut. *Microbiol Resour Announc* 2019;**8**:10-1128.
2. Chklovski A, Parks DH, Woodcroft BJ *et al.* CheckM2: A rapid, scalable and accurate tool for assessing microbial genome quality using machine learning. *Nat Methods* 2023;**20**:1203–12.
3. Kawano K, Ushijima N, Kihara M *et al. Patiriisocius marinistellae* gen. nov., sp. nov., isolated from the starfish *Patiria pectinifera*, and reclassification of *Ulvibacter marinus* as a member of the genus *Patiriisocius* comb. nov. *Int J Syst Evol Microbiol* 2020;**70**:4119–29.
4. Parks DH, Chuvochina M, Rinke C *et al.* GTDB: an ongoing census of bacterial and archaeal diversity through a phylogenetically consistent, rank normalized and complete genome-based taxonomy. *Nucleic Acids Res* 2022;**50**:D785–94.
5. Ma B, Lu C, Wang Y *et al.* A genomic catalogue of soil microbiomes boosts mining of biodiversity and genetic resources. *Nat Commun* 2023;**14**:7318.
6. Sereika M, Mussig AJ, Jiang C *et al.* Genome-resolved long-read sequencing expands known microbial diversity across terrestrial habitats. *Nat Microbiol* 2025;**10**:2018–30.
7. Chaumeil P-A, Mussig AJ, Hugenholtz P *et al.* GTDB-Tk v2: memory friendly classification with the genome taxonomy database. *Bioinform* 2022;**38**:5315–16.
8. Hyatt D, Chen G-L, LoCascio PF *et al.* Prodigal: prokaryotic gene recognition and translation initiation site identification. *BMC Bioinform* 2010;**11**:119.
9. Haft DH, Loftus BJ, Richardson DL *et al.* TIGRFAMs: a protein family resource for the functional identification of proteins. *Nucleic Acids Res* 2001;**29**:41–43.
10. Aramaki T, Blanc-Mathieu R, Endo H *et al.* KofamKOALA: KEGG ortholog assignment based on profile HMM and adaptive score threshold. *Bioinform* 2020;**36**:2251–52.
11. Eddy SR, Wheeler T. HMMER: Biosequence analysis using profile hidden Markov models. http://hmmer.org 2007
12. Katoh K, Misawa K, Kuma Ki *et al.* MAFFT: a novel method for rapid multiple sequence alignment based on fast Fourier transform. *Nucleic Acids Res* 2002;**30**:3059–66.
13. Price MN, Dehal PS, Arkin AP. FastTree 2–approximately maximum-likelihood trees for large alignments. *PLoS ONE* 2010;**5**:e9490.
14. Mise K, Masuda Y, Senoo K *et al.* Undervalued pseudo-*nifH* sequences in public databases distort metagenomic insights into biological nitrogen fixers. *mSphere* 2021;**6**:e00785-21.
15. Vaser R, Pavlović D, Šikić M. SWORD—a highly efficient protein database search. *Bioinform* 2016;**32**:i680–84.
16. Junier T, Zdobnov EM. The Newick utilities: high-throughput phylogenetic tree processing in the UNIX shell. *Bioinform* 2010;**26**:1669–70.
17. Masuda Y, Mise K, Xu Z *et al.* Global soil metagenomics reveals distribution and predominance of *Deltaproteobacteria* in nitrogen-fixing microbiome. *Microbiome* 2024;**12**:95.

18. Mise K, Masuda Y, Itoh H. Quality-filtered soil shotgun metagenomes. https://doi.org/10.25452/figshare.plus.25332547 (2024)

19. Huerta-Cepas J, Szklarczyk D, Heller D *et al.* eggNOG 5.0: a hierarchical, functionally and phylogenetically annotated orthology resource based on 5090 organisms and 2502 viruses. *Nucleic Acids Res* 2019;**47**:D309–14.

20. Buchfink B, Reuter K, Drost HG. Sensitive protein alignments at tree-of-life scale using DIAMOND. *Nat Methods* 2021;**18**:366–68.

21. Mise K, Masuda Y, Senoo K *et al.* Betaproteobacterial clade II *nosZ* activated under high N_2O concentrations in paddy soil microcosms. *J Appl Microbiol* 2025;**136**:lxaf055.

22. Matsen FA, Kodner RB, Armbrust EV. pplacer: linear time maximum-likelihood and Bayesian phylogenetic placement of sequences onto a fixed reference tree. *BMC Bioinform* 2010;**11**:538.

23. Mise K, Wasai-Hara S, Itoh H. Global terrestrial distribution of N_2O-reducing *Acidobacteriota* members. *ISME J* 2026;wrag073.

24. Woodcroft BJ, Singleton CM, Boyd JA *et al.* Genome-centric view of carbon processing in thawing permafrost. *Nature* 2018;**560**:49–54.

25. Rognes T, Flouri T, Nichols B *et al.* VSEARCH: a versatile open source tool for metagenomics. *PeerJ* 2016;**4**:e2584.

26. Letunic I, Bork P. Interactive Tree of Life (iTOL) v6: recent updates to the phylogenetic tree display and annotation tool. *Nucleic Acids Res* 2024;**52**:W78–82.

27. Shen W, Le S, Li Y *et al.* SeqKit: A cross-platform and ultrafast toolkit for FASTA/Q file manipulation. *PLoS One* 2016;**11**:e0163962.

28. Shen W, Ren H. TaxonKit: A practical and efficient NCBI taxonomy toolkit. *J Genet Genom* 2021;**48**:844–50.

29. Zhang Q, Fu J, Chen Z *et al*. *Paracidobacterium acidisoli* gen. nov., sp. nov. and *Alloacidobacterium dinghuense* gen. nov., sp. nov., two *Acidobacteria* isolated from forest soil, and reclassification of *Acidobacterium ailaaui* and *Acidipila dinghuensis* as *Pseudacidobacterium ailaaui* gen. nov., comb. nov. and *Silvibacterium dinghuense* comb. nov. *Int J Syst Evol Microbiol* 2022;**72**:005415.

30. Kishimoto N, Kosako Y, Tano T. *Acidobacterium capsulatum* gen. nov., sp. nov.: An acidophilic chemoorganotrophic bacterium containing menaquinone from acidic mineral environment. *Curr Microbiol* 1991;**22**:1–7.

31. Lladó S, Benada O, Cajthaml T *et al*. *Silvibacterium bohemicum* gen. nov. sp. nov., an acidobacterium isolated from coniferous soil in the bohemian forest national park. *Syst Appl Microbiol* 2016;**39**:14–19.

32. Okamura K, Kawai A, Yamada T *et al*. *Acidipila rosea* gen. nov., sp. nov., an acidophilic chemoorganotrophic bacterium belonging to the phylum *Acidobacteria*. *FEMS Microbiol Lett* 2011;**317**:138–42.

33. Belova SE, Ravin NV, Pankratov TA et al. Hydrolytic capabilities as a key to environmental success: chitinolytic and cellulolytic *Acidobacteria* from acidic sub-arctic soils and boreal peatlands. *Front Microbiol* 2018;**9**:2775.

34. Ou-Yang T, Xia F, Qiu L. *Acidicapsa dinghuensis* sp. nov., a novel acidobacterium isolated from forest soil. *Int J Syst Evol Microbiol* 2018;**68**:2364–69.

35. García-Fraile P, Benada O, Cajthaml T *et al*. *Terracidiphilus gabretensis* gen. nov., sp. nov., an abundant and active forest soil acidobacterium important in organic matter transformation. *Appl Environ Microbiol* 2016;**82**:560–69.

36. Foesel BU, Mayer S, Luckner M *et al*. *Occallatibacter riparius* gen. nov., sp. nov. and *Occallatibacter savannae* sp. nov., *Acidobacteria* isolated from Namibian soils, and emended description of the family *Acidobacteriaceae*. *Int J Syst Evol Microbiol* 2016;**66**:219–29.

37. Eichorst SA, Breznak JA, Schmidt TM. Isolation and characterization of soil bacteria that define *Terriglobus* gen. nov., in the phylum *Acidobacteria*. *Appl Environ Microbiol* 2007;**73**:2708–17.

38. Gerhardt K, Ruiz-Perez CA, Rodriguez-R LM, Jain C, Tiedje JM, Cole JR, Konstantinidis KT. FastAAI: efficient estimation of genome average amino acid identity and phylum-level relationships using tetramers of universal proteins. *Nucleic Acids Res* 2025;**53**:gkaf348.

39. Tripathi AK, Nagarajan T, Verma SC *et al.* Inhibition of biosynthesis and activity of nitrogenase in *Azospirillum brasilense* Sp7 under salinity stress. *Curr Microbiol* 2002;**44**:363–67.

40. Kuykendall L, Elkan G. *Rhizobium japonicum* derivatives differing in nitrogen-fixing efficiency and carbohydrate utilization. *Appl Environ Microbiol* 1976;**32**:511–19.

41. Srivastava S, Dong H, Baars O *et al.* Bioavailability of mineral-associated trace metals as cofactors for nitrogen fixation by *Azotobacter vinelandii*. *Geobiol* 2023;**21**:507–19.

42. Wertz JT, Kim E, Breznak JA *et al.* Genomic and physiological characterization of the *Verrucomicrobia* isolate *Diplosphaera colitermitum* gen. nov., sp. nov., reveals microaerophily and nitrogen fixation genes. *Appl Environ Microbiol* 2012;**78**:1544–55.

43. Masuda Y, Yamanaka H, Xu Z-X *et al.* Diazotrophic *Anaeromyxobacter* isolates from soils. *Appl Environ Microbiol* 2020;**86**:e00956-20.

44. Jing X, Liu X, Zhang Z *et al.* Anode respiration-dependent biological nitrogen fixation by *Geobacter sulfurreducens*. *Water Res* 2022;**208**:117860.

45. Chen J, Toth J, Kasap M. Nitrogen-fixation genes and nitrogenase activity in *Clostridium acetobutylicum* and *Clostridium beijerinckii*. *J Ind Microbiol Biotechnol* 2001;**27**:281–86.

46. Kucho K, Tamari D, Matsuyama S *et al.* Nitrogen fixation mutants of the actinobacterium *Frankia casuarinae* CcI3. *Microbes Environ* 2017;**32**:344–51.
